# Record-High Efficiency Blue-Green Cationic Ir(III) Complexes for Light-Emitting Electrochemical Cells with EQE Approaching 40%

**DOI:** 10.1021/acs.inorgchem.5c00167

**Published:** 2025-05-19

**Authors:** Yu-Ting Huang, Chung-Chieh Chang, Che-Lun Chang, Wei-Tse Hsu, Yun-Rong Li, Zu-Po Yang, Chin-Wei Lu, Hai-Ching Su

**Affiliations:** † Department of Applied Chemistry, 63293 Providence University, Taichung 43301, Taiwan; ‡ Institute of Lighting and Energy Photonics, 529897 National Yang Ming Chiao Tung University, Tainan 71150, Taiwan; § Institute of Photonic System, National Yang Ming Chiao Tung University, Tainan 71150, Taiwan

## Abstract

Light-emitting electrochemical cells (LECs) provide a cost-effective solution for lighting applications and are well-suited for large-area and industrial-scale manufacturing. However, enhancing the efficiency of LECs remains a significant challenge. To address this issue, this study presents a series of blue-green iridium complexes with promising phosphorescent emission properties. Among these, di­[1-(2,4-difluorophenyl)-pyrazolyl]-5,5′-difluoro-2,2′-bipyridyl iridium­(III) hexafluorophosphate stands out, demonstrating exceptional performance. Following optimizing the device with varying thicknesses, an EQE of 16.8% and a current efficiency of 47.2 cd A^–1^ were attained. Further enhancements through the integration of a diffusive layer resulted in a 270% increase in efficiency, reaching an EQE of 39.3% and current efficiency of 109.6 cd A^–1^. This efficient technology demonstrates significant potential and lays the groundwork for future high-performance light-emitting devices.

## Graphical abstract

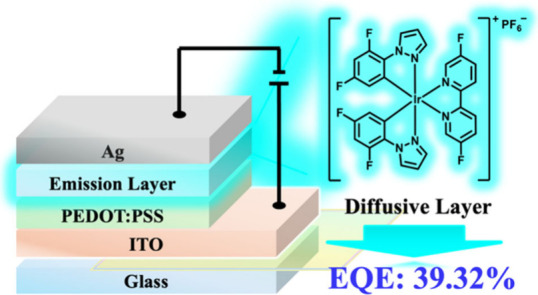

## Introduction

In recent years, organic light-emitting diodes (OLEDs) have attained significant success in lighting and displays, becoming widely known and used in our daily lives. Concurrently, they emerge as a popular and rapidly developing subject, with thousands of compounds reported.^1–4^ However, the multilayer structure of organic light-emitting diodes (OLEDs) requires a complex and costly manufacturing process, including vacuum deposition and strict encapsulation to prevent degradation.^5–7^ In contrast, Pei et al. first demonstrated the concept of light-emitting electrochemical cells (LECs) with a simpler structure in 1995.^8^ They utilize low work function metal electrodes (such as aluminum or silver) and solution-processed techniques (like spin coating and roll-to-roll methods), which simplify fabrication and improve cost-effectiveness.

Statistics indicate that urban lighting applications alone consume 20% of the world’s total electricity.^9–11^ As a result, the improvement of component efficiency and the reduction of energy consumption in lighting devices have become urgent priorities. Light-emitting electrochemical cells (LECs) are gradually being recognized as promising candidates for next-generation lighting and display technologies. LECs are a type of simple electroluminescent device, consisting of an ionic active layer sandwiched between two air-stabile electrodes. The luminescent materials used in LECs generally include conjugated polymers,^12–16^ quantum dots (QDs),^17–20^ perovskites,^21–23^ small molecules,^24–27^ and ionic transition metal complexes (iTMCs)^28–32^ have been proposed. In the electroluminescence process, iTMCs containing elements such as iridium (Ir),^33–47^ ruthenium (Ru),^48–51^ and platinum (Pt)^52–55^ exhibit strong spin–orbit coupling (SOC), which significantly enhances intersystem crossing (ISC) from singlet to triplet excitons. This mechanism results in the maximization of internal quantum efficiency (IQE), enabling a theoretical exciton utilization rate of 100%.

Among all iTMCs utilized in LECs, Ir­(III) iTMCs have demonstrated exceptional performance, attributed to their short triplet lifetimes and high phosphorescence quantum yields. Typical cationic cyclometalated Ir­(III) complexes with the general represented by [Ir­(C^∧^N)_2_(N^∧^N)^+^(PF_6_)^−^], allowing for tunable emission colors across the entire visible spectrum by controlling the C^∧^N and N^∧^N ligands. In the design of blue-emitting complexes, the commonly used incorporation of five-membered nitrogen-rich heterocycles (such as pyrazole or triazole) and electron-withdrawing groups (e.g., fluorine (−F), trifluoromethyl (−CF_3_), or cyano (−CN)) into the C^∧^N ligands contributes to highest occupied molecular orbital (HOMO) stabilization, thereby resulting in blue-shifted emission and enhanced photostability. Based on this design philosophy, in 2021, He et al. reported a blue-green emitting complex [Ir­(CF_3_-dPhTAZ)_2_(bpy)]­PF_6_, incorporating a phenyl-triazole type C^∧^N ligand to construct highly efficient cationic iridium complexes.^56^ This modification resulted in blue-shifted emission and suppressed phosphorescence concentration quenching, with an EQE of 10.4% at 525 nm. Subsequently, Lu et al. utilized a novel C^∧^N ligand with long π-conjugation systems containing – CN and – F groups to enhance molecular polarity.^57^ Ultimately, devices containing the diffusive layer achieved EQE of 22.15% at 532 nm. Based on our previous work, the introduction of electron-withdrawing fluorine atoms into the C^∧^N ligand, along with methyl substitution on the N^∧^N ligand, resulted in an increased band gap (*E*_g_) and commendable photoluminescence quantum yield (PLQY).^58^ After the embedding of a diffusive layer on the substrate, the optimized EQE was measured at 35.4%, demonstrating the best performance observed in blue-green LECs at that time. However, the carrier balance remains imperfect and developing more efficient Ir-LECs to enhance electroluminescent performance still presents significant challenges, warranting further exploration.

In this study, inspired by previous findings and insights, 1-(2,4-difluorophenyl)-pyrazole (**dfppz**) was chosen as the C^∧^N ligand. It is known that F-substituents on the C^∧^N ligand can stabilize the HOMO, typically leading to a blue shift and enhanced photoluminescence quantum yield (PLQY). In addition, a fluorination strategy for the N^∧^N ligand was also employed, specifically using F-substituted bipyridine (**dfbp**). On the other hand, methoxyl (−OCH_3_) groups were introduced to N^∧^N ligand suppress quenching behavior induced by intermolecular π-π interactions (**fomp** and **domp**). Among them, di­[1-(2,4-difluorophenyl)-pyrazolyl]-5,5′-difluoro-2,2′-bipyridyl iridium­(III) hexafluorophosphate (**DFBP**) has a PLQY of up to 76%, and the EQE of the LECs reaches 16.8%. Furthermore, by incorporating a diffusive layer composed of TiO_2_ nanoparticles (NPs), a record-high EQE of 39.3% was achieved. This breakthrough marks a milestone in the development of next-generation high-efficiency electroluminescent devices.

## Experimental Section

### General Information

All reactants and solvents were purchased from commercial sources such as Aldrich, Acros, or Fluorochem. Cyclometalated dinuclear iridium complex with the general formula (C^∧^N)_2_Ir­(μ-Cl)_2_Ir­(C^∧^N)_2_ were synthesized using established procedures from the literature.^57,59^ Complexes were structurally elucidated using ^1^H NMR and ^13^C NMR spectroscopy, ESI-Mass spectrum and elemental analysis. Nuclear magnetic resonance (NMR) spectra of the compounds were collected using a Bruker Ascend 400 MHz spectrometer at room temperature. The photophysical characteristics of all complexes were measured at room temperature using an Edinburgh FS5 spectrofluorometer, with 1 × 10^–5^ M acetonitrile (MeCN) solutions prepared for analysis. UV–vis absorption spectra were obtained using PerkinElmer Lambda 14 spectrophotometer. To further gain properties of these complexes, complete geometry optimizations were performed using density functional theory (DFT) with the B3LYP/LANL2DZ­[Ir]­6–31G­(d,p)­[F,O,N,C,H] basis set in Gaussian 09. Furthermore, time-dependent DFT (TD-DFT) and the unrestricted B3LYP (UB3LYP) method were employed to investigate the excited-state characteristics, providing a more comprehensive theoretical analysis. The oxidation and reduction potentials of all complexes were measured via cyclic voltammetry (CV) at a scan rate of 100 mV s^–1^ in MeCN solution (3 × 10^–4^ M) on CHI 611E. A glassy carbon electrode and platinum wire were used as the working electrode and the counter electrode, respectively. All potentials were recorded relative to an Ag/AgCl (saturated) reference electrode. A solution of 0.1 M tetrabutylammonium hexafluorophosphate (TBAPF_6_) in MeCN solution was employed as the supporting electrolyte.

### Synthetic Procedures

#### Synthesis of Di­[1-(2,4-difluorophenyl)-pyrazoyl]-5,5′-difluoro-2,2′-bipyridyl iridium­(III) hexafluorophosphate (**DFBP**)

The cyclometalated dichloro-bridged dimeric iridium [Ir­(dppfz)_2_Cl]_2_ (0.39 g, 0.33 mmol) and 5,5′-difluoro-2,2′-bipyridine (**dfbp**) (0.18 g, 0.66 mmol), were dissolved in degassed methanol (40 mL). The reaction mixture was heated to reflux at 80 °C for 24 h under nitrogen atmosphere After cooling to room temperature, the solution was poured into KPF_6_ aqueous solution and stirred for 1.5 h. It was then diluted with 100 mL of dichloromethane (DCM), and the solvent was removed under reduced pressure. The crude product was purified by column chromatography using DCM/methanol (50/3 to 50/2, v/v) as the eluent, followed by recrystallizations from DCM/*n*-hexane, yielding **DFBP** as a bright green solid (0.48 g, yield: 82%). ^1^H NMR (400 MHz, CD_3_CN): δ 8.51 (dd, *J* = 9.2, 4.4 Hz, 2H), 8.45 (d, *J* = 2.9 Hz, 2H), 7.02–7.97 (m, 2H), 7.95 (t, *J* = 2.2 Hz, 2H), 7.14 (d, *J* = 2.4 Hz, 2H), 6.89–6.80 (m, 2H), 6.64 (t, 2.9 Hz, 2H), 5.73 (dd *J* = 8.6, 2.4 Hz, 2H). ^13^C NMR (100 MHz, CD_3_CN): δ 163.3, 162.3, 162.2, 160.7, 159.8, 159.7, 153.0, 151.4, 151.3, 148.9, 148.8, 141.7, 141.4, 140.2, 136.3, 133.5, 133.3, 128.4, 128.2, 128.0, 127.4, 116.18, 116.15, 115.98, 115.95, 109.7, 100.8, 100.51, 100.47, 100.2. HRMS (ESI^+^) *m*/*z*: calcd for C_28_H_16_F_6_N_6_Ir^+^ [M–PF_6_]^+^: 743.0964, found: 743.0970 Anal. Calcd for C_28_H_16_F_12_IrN_6_P: C, 37.89; H, 1.82; N, 9.47, found C, 37.59; H, 2.22; N, 9.48.

#### Synthesis of Di­[1-(2,4-difluorophenyl)-1H-pyrazoyl]-5-fluoro-5′-methoxy-2,2′-bipyridyl iridium­(III) hexafluorophosphate (**FOMP**)

Following the procedure for **DFBP**, [Ir­(dppfz)_2_Cl]_2_ and 5-fluoro-5′-methoxy-2,2′-bipyridine (**fomp**) gave **FOMP** as a green solid (0.42 g, yield: 70%). ^1^H NMR (400 MHz, CD_3_CN) δ 8.44–8.37 (m, 4H), 7.97–7.90 (m, 2H), 7.72 (dd, *J* = 8.8, 2.8 Hz, 1H), 7.65 (d, *J* = 2.8 Hz, 1H), 7.14 (d, *J* = 2.4 Hz, 1H), 7.10 (d, *J* = 2.2 Hz, 1H), 6.85–6.79 (m, 2H), 6.63–6.61 (m, 2H), 5.77–5.73 (m, 2H), 3.82 (s, 3H).^13^C NMR (100 MHz, CD_3_CN): δ 162.8, 162.5, 162.4, 162.3, 160.2, 159.9, 159.8, 154.20, 154.16, 151.5, 151.4, 151.3, 149.00, 148.93, 148.8, 148.5, 141.2, 141.0, 140.8, 140.13, 140.07, 137.4, 137.2, 133.53, 133.49, 133.39, 133.35, 128.5, 128.2, 128.0, 126.8, 126.3, 126.2, 124.4, 116.3, 116.3, 116.1, 115.93, 115.90, 109.8, 100.69, 100.65, 100.5, 100.42, 100.37, 100.2, 100.1, 57.3. HRMS (ESI^+^) *m*/*z*: calcd for C_29_H_19_F_5_N_6_OIr^+^ [M–PF_6_]^+^: 755.1164, found: 755.1164. Anal. Calcd for C_29_H_19_F_11_IrN_6_OP: C, 38.72; H, 2.13; N, 9.34, found C, 38.36; H, 1.97; N, 9.12.

#### Synthesis of 5,5′-Dimethoxy-2,2′-bipyridyl-di­[1-(2,4-difluorophenyl)-1H-pyrazoyl] iridium­(III) hexafluorophosphate (**DOMP**)

Following the procedure for **DFBP**, the reaction of [Ir­(dppfz)_2_Cl]_2_ and 5,5′-dimethoxy-2,2′-bipyridine (**domp**) yielded **DOMP** as a green solid (0.48 g, yield: 80%). ^1^H NMR (400 MHz, CD_3_CN): δ 8.44 (d, *J* = 4.0 Hz, 2H), 8.31 (dd, *J* = 10.4, 4.0 Hz, 2H), 7.70 (d, *J* = 9.2 Hz, 2H), 7.63 (s, 2H), 7.11 (s, 2H), 6.84–6.78 (m, 2H), 6.63 (t, *J* = 2.8 Hz, 2H), 5.78 (dd, *J* = 8.2, 2.8 Hz, 2H), 3.81 (s, 6H). ^13^C NMR (100 MHz, CD_3_CN): δ 162.3, 162.2, 159.8, 159.7, 159.0, 151.3, 151.2, 149.5, 148.88, 148.7, 140.0, 139.8, 138.0, 137.9, 133.3, 133.2, 128.39, 128.35, 125.4, 124.5, 116.03, 116.00, 115.83, 115.80, 109.63, 109.60, 100.4, 100.2, 100.1, 100.0, 57.1. HRMS (ESI^+^) *m*/*z*: calcd for C_30_H_22_F_4_N_6_O_2_Ir^+^ [M–PF_6_]^+^: 767.1364, found: 767.1358. Anal. Calcd for C_30_H_22_F_10_IrN_6_O_2_P: C, 39.52; H, 2.43; N, 9.22, found C, 39.43; H, 2.90; N, 9.72.

### Fabrication of Diffusive Substrates

The diffuser film was made of a transparent photoresist (TPR) layer doped with 250 and 25 nm titanium dioxide nanoparticles (TiO_2_ NPs). The TPR (EOC170) was sourced from Everlight Chemical Industrial Corporation. TiO_2_ NPs were subjected to ultrasonic shaking in the TPR solution for 24 h. The weights of the TPR solution, 250 nm TiO_2_ NPs, and 25 nm TiO_2_ NPs were 4.0, 0.6, and 1.1 g, respectively. The uniformly mixed TPR/NP solutions were filtered and then spin-coated onto the glass substrates. The spin-coated diffuser films were baked at 100 °C for 7 min, followed by 200 °C for 10 min. The thickness of the diffuser film, measured by a surface profiler, was approximately 1.8 μm. Finally, the indium tin oxide (ITO) films (160 nm) were deposited on the diffusive substrates by DC sputtering.

### Device Fabrication and Characterization

The device fabrication process began with a standard cleaning procedure, followed by UV/ozone treatment on glass or diffusive substrates coated with ITO layers. After cleaning, the substrates were spin-coated with a 40 nm layer of poly­(3,4-ethylenedioxythiophene):poly­(styrenesulfonate) (PEDOT:PSS) at 3500 rpm, then baked at 150 °C for 30 min in ambient air. A solution mixture containing 80 wt % of complexes and 20 wt % of 1-butyl-3-methylimidazolium hexafluorophosphate [BMIM^+^(PF_6_)^−^] in MeCN was spin-coated onto the PEDOT:PSS layer. The incorporation of the ionic liquid [BMIM^+^(PF_6_)^−^] aimed to introduce additional mobile ions, thereby enhancing the device response. Various solution concentrations were used during spin coating to achieve different thicknesses of the emissive layers, aiming to optimize device performance. The emissive layers were spin-coated at 1500 or 1000 rpm for 60 s in ambient air. Ellipsometry was utilized to measure the thickness of the emissive layer. After depositing the emissive layers, the samples were placed in a vacuum oven at 60 °C for 8 h to remove remaining solvent. Finally, a silver top contact was deposited using thermal evaporation in a vacuum chamber with a pressure of approximately 10^–6^ Torr. The EL emission properties of these LECs were assessed using source-measurement units (B2901A, Keysight) in conjunction with a calibrated Si photodiode. The EL spectra of these LECs were recorded using a calibrated fiber-optic spectrometer (USB2000, Ocean Optics). All LECs devices were tested under constant bias voltages, with measurements conducted in a nitrogen glovebox to minimize device degradation.

## Results and Discussion

### Synthesis and Structural Characterization

A series of pyridine-based N^∧^N ligands (**dfbp**, **fomp**, and **domp**) were prepared according to the literature.^57,60^ The synthetic route for C^∧^N ligand **dfppz** and all targeted complexes **DFBP**, **FOMP**, and **DOMP** are shown in Scheme 1. Initially, (2,4-difluorophenyl) hydrazinium chloride and 1,1,3,3-tetramethoxypropane via condensation to obtain 1-(2,4-difluorophenyl)-pyrazole (**dfppz**, 86%). Thereafter, C^∧^N ligand **dfppz** and iridium­(III) chloride hydrate are heated under reflux in a mixed solvent of 2-ethoxyethanol and water (v/v = 3:1), resulting in the precipitation of the chloride-bridged iridium dimer [Ir­(dfppz)_2_Cl]_2_. Finally, [Ir­(dfppz)_2_Cl]_2_ are reacted with 2 equiv N^∧^N ligand in methanol followed by ion exchange to PF_6_, the targeted complexes are isolated through precipitation. All complexes were purified by silica gel column chromatography and subsequently recrystallized from DCM /*n*-hexane solvent. These complexes exhibit high solubility in DCM, MeCN, and dimethyl sulfoxide (DMSO), but their solubility is quite limited in nonpolar solvents such as *n*-hexane and toluene. These iridium complexes were identified by ^1^H NMR, ^13^C NMR spectroscopy, mass spectrometry, and elemental analysis. The characterization data for these products are available in the Supporting Information.

**Scheme 1:**
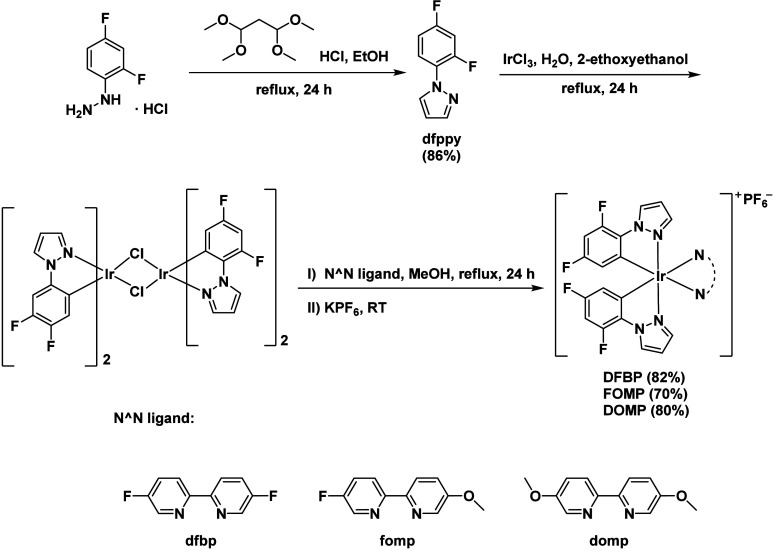
Synthetic Routes for Targeted Complexes

### Photophysical Properties

The photophysical properties of these complexes were analyzed ultraviolet–visible (UV–vis) absorption and photoluminescence (PL) spectra in MeCN solution (1.0 × 10^–5^ M) at room temperature, as shown in Table 1. The strongest absorption peaks at 200–300 nm range correspond to the spin-allowed π → π* transitions of the C^∧^N and N^∧^N ligands.^57,61^ The weak absorption peaks at 300–350 nm are assigned to the spin-allowed metal-to-ligand charge transfer (MLCT) and ligand-to-ligand charge transfer (LLCT) transitions, while the further weaker absorption peaks in the 350–450 nm observed due to the spin-prohibited MLCT, LLCT, and ligand-centered (LC) transitions.^62–64^ As seen in Figure 1, the complexes show emission peaks between 476 and 544 nm, which are indicative of blue-green photoluminescence. Moreover, **DFBP** exhibits a broad and featureless spectrum, indicating an emission related to MLCT with a peak at 517 nm. In contrast, **FOMP** and **DOMP** display vibronically structured peaks with emissions centered at 508 nm (476, 531 nm (sh.)) and 522 nm (488, 544 nm (sh.)), respectively. These structured emission peaks are attributed to the combination of LC π → π*, LLCT, and MLCT characteristics in the excited state, as confirmed by TD-DFT calculations on the optimized S_0_ geometry (Table S1) and natural transition orbitals (NTOs) analysis (Figure 6). Additionally, the shoulder peaks (onset) of **DFBP**, **FOMP**, and **DOMP** in MeCN solution are 440, 445, and 456 nm, respectively. The red shift of emission wavelengths with the increase in methoxy groups indicates an extension of the conjugation system. To further investigate the photophysical properties of these complexes, transient photoluminescence (TrPL) and photoluminescence quantum yield (PLQY) measurements were conducted. The TrPL spectra are provided in the Supporting Information, and the excited-state lifetimes obtained from the TrPL profiles are summarized in (Table 1). The excited-state lifetime can be fitted by a single-exponential decay (τ) range from 1.97 to 16.81 μs, confirming the phosphorescent properties of the emissions. The PLQY values for **DFBP**, **FOMP**, and **DOMP** are 76, 64, and 30%, respectively, consistent with previous reports indicating that highly fluorinated complexes often exhibit high quantum yields, whereas methoxy-substituted **FOMP** and **DOMP** have lower quantum yields due to the decreased rigidity. Based on τ and PLQY measurements, the radiative rate constants (*k*_r_) and nonradiative rate constants (*k*_*n*r_) for targeted complexes were determined. **DFBP**, **FOMP**, and **DOMP** exhibit *k*_r_ values of 4.84 × 10^5^, 8.77 × 10^5^, and 0.45 × 10^5^ s^–1^, respectively. Although **FOMP** shows a slightly faster *k*_r_ than **DFBP**, its corresponding *k*_nr_ is also significantly higher, resulting in a lower PLQY. The delicate balance between radiative and nonradiative processes plays a key role in determining the PLQY.

**Figure 1:**
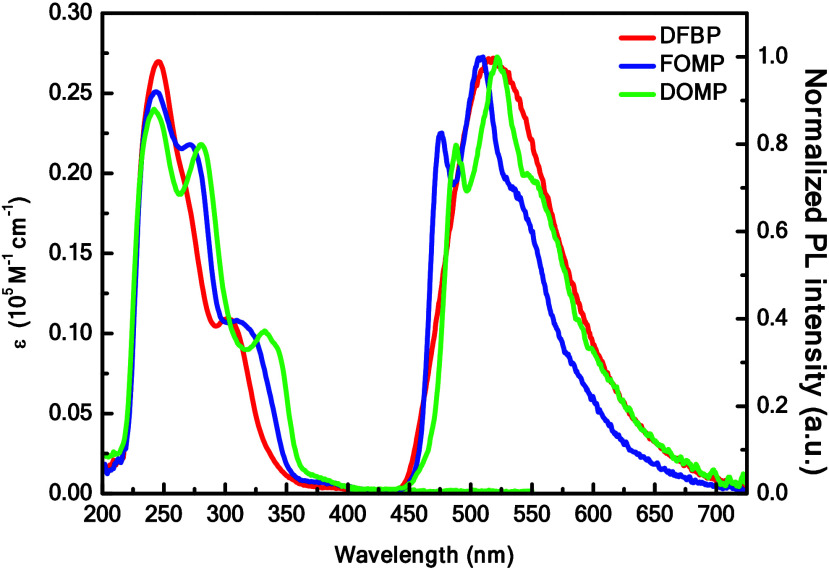
Schematic representation of absorption (left axis) and PL spectra (right axis) of **DFBP**, **FOMP**, and **DOMP** in MeCN solution.

**Table 1:** Photophysical characteristics of targeted complexes

	MeCN solution						thin film doped with 20 wt % [BMIM^+^(PF_6_)^−^]				
Complex	λ_abs_ (nm)^a^	λ_em_ (nm)^a^	PLQY (%)^a^	τ (μs)^a^	*k*_r_ (10^5^ s^–1^)^c^	*k*_nr_ (10^5^ s^–1^)^c^	λ_em_ (nm)^b^	PLQY (%)^b^	τ (μs)^b^	*k*_r_ (10^5^ s^–1^)^c^	*k*_nr_ (10^5^ s^–1^)^c^
DFBP	245, 305	517	76	1.57	4.84	1.53	499	69	1.41	4.90	2.20
FOMP	245, 269, 314	476, 508, 544	64	0.73	8.77	4.93	481, 511	60	8.27	0.73	0.48
DOMP	245, 280, 311	488, 522, 544	31	6.92	0.45	0.10	487, 524	30	15.5	0.19	0.45

a Measured in MeCN solution (1.0 × 10^–5^ M) at RT.

b Measured in thin film doped with 20 wt % [BMIM^+^(PF_6_)^−^] at RT.

c Estimated based on the equations *k*_r_ = PLQY × τ^–1^ and *k*_nr_ = (1 – PLQY) × τ^–1^.

For evaluating the potential use of the complexes in light-emitting devices, emission properties of the complexes in thin films doped with 20 wt % [BMIM^+^(PF_6_)^−^]. Table 1 summarizes detailed Photophysical characteristics in thin films. The emission maxima for **DFBP**, **FOMP**, and **DOMP** were observed at 499, 511, and 524 nm, respectively, showing a gradual shift to longer wavelengths across the complexes. The PLQY for **DFBP**, **FOMP**, and **DOMP** are 69, 60, and 30%, respectively, slightly lower than in solution, likely due to exciton quenching in the condensed molecular films. The *k*_r_ value for **DFBP** is 4.90 × 10^5^ s^–1^, significantly higher than those of **FOMP** and **DOMP** (*k*_r_ = 0.73 × 10^5^ s^–1^ and 0.19 × 10^5^ s^–1^, respectively). This further indicates that the reduction in MLCT characteristics in **FOMP** and **DOMP** highlights the substantial LC π → π* features in their photophysical behavior.^65^

### Electrochemical and Thermal Properties

The electrochemical properties were studied by cyclic voltammetry (CV) measurements in MeCN solution (3 × 10^–4^ M) solution using ferrocene as an internal reference (Figure 2 and Table 2).^9,64^ The oxidation and reduction potentials (determined from the onset peak) of this series of complexes exhibit minimal variation, with oxidation potentials ranging from 1.22 to 1.25 eV and reduction potentials from −1.16 to −1.18 eV. Using the equations *E*_HOMO_ = −4.8 + [*E*_*1/2*ox_ (Fc) – *E*_ox_] and *E*_LUMO_ = −4.8 + [*E*_*1/2*ox_ (Fc) – *E*_re_], the corresponding electrochemical energy gaps were 2.43, 2.40, and 2.38 eV, respectively, which are consistent with the optical energy gaps. To further explore their potential applications in LECs, thermal stability was evaluated through thermogravimetric analysis (TGA) for targeted complexes, as shown in Figure 3. The decomposition temperatures (*T*_d_), i.e., the temperature at which a weight loss of 5% was recorded, for **DFBP**, **FOMP**, and **DOMP** were found to be 294, 302, and 324 °C, respectively, indicating their potential for LECs applications. It is well established that in OLEDs, a higher number of *sp* ^2^ C–F bonds is typically associated with a reduction in *T*_d_, which may negatively impact device stability.^66^ However, in the case of LECs: (1) devices are generally solution-processed, thereby avoiding the high temperatures required for vacuum deposition in OLEDs that can lead to thermal degradation; and (2) the inclusion of additional *sp* ^2^ C–F bonds has, in fact, been reported to enhance device stability.^67,68^ Therefore, although a decrease in *T*_d_ may occur upon their incorporation, the overall impact on LECs is generally limited and can even be advantageous in certain scenarios.

**Figure 2:**
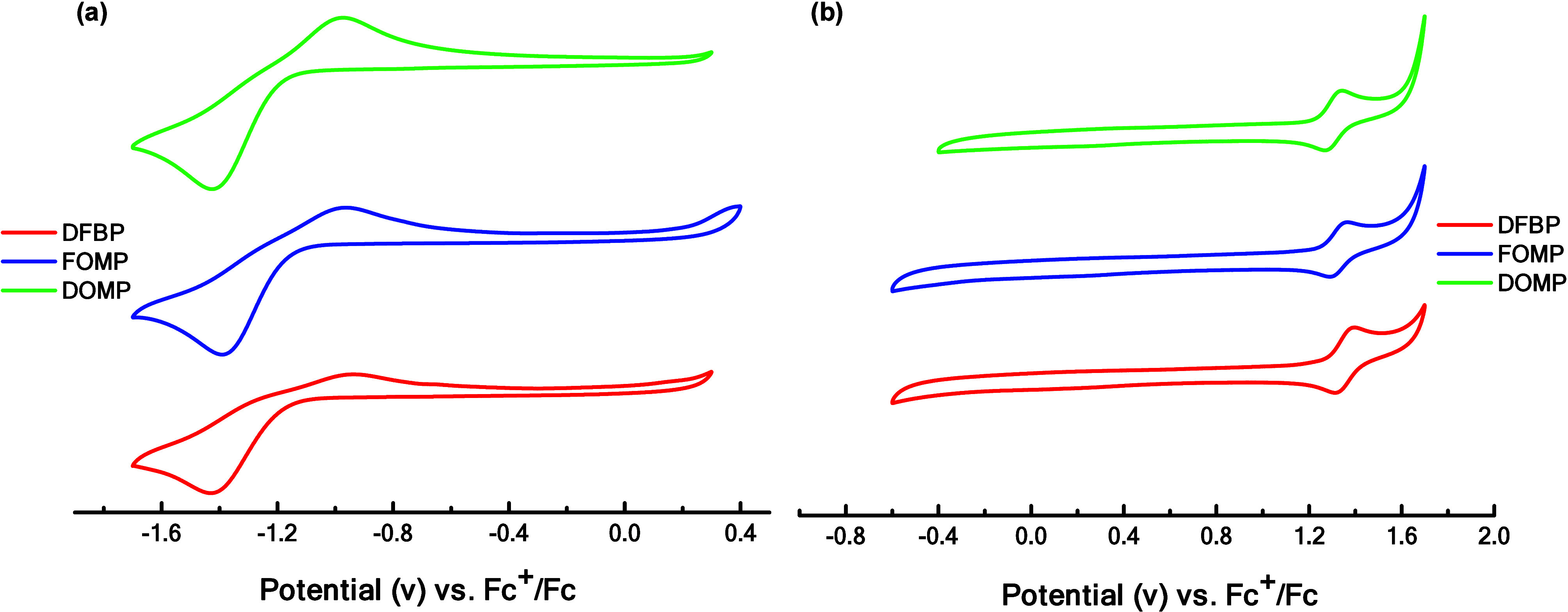
Cyclic voltammograms of (a) reduction and (b) oxidation processes of the targeted complexes in MeCN. Potentials were recorded vs Fc^+^/Fc.

**Table 2:** Selected physical data of targeted complexes

Complex	*E*_g_^opt^ (eV)^a^	*E*_ox_ (V)^b^	*E*_re_ (V)^b^	*E*_HOMO_ (eV)^c^	*E*_LUMO_ (eV)^d^	*E*_g_ (eV)^e^
**DFBP**	2.81	1.25	–1.18	–5.50	–3.07	2.43
**FOMP**	2.79	1.24	–1.16	–5.49	–3.09	2.40
**DOMP**	2.72	1.22	–1.16	–5.47	–3.09	2.38

a Estimated from the onsets of emission spectra 1 × 10^–5^ M MeCN solution at RT.

b TBAPF_6_ as the electrolyte (0.1 M MeCN solution). Potential vs ferrocene/ferrocenium redox couple.

c Estimated from the half-wave oxidation potentials.

d Estimated from the half-wave reduction potentials.

e *E*_g_ = *E*_LUMO_ – *E*_HOMO_.

**Figure 3:**
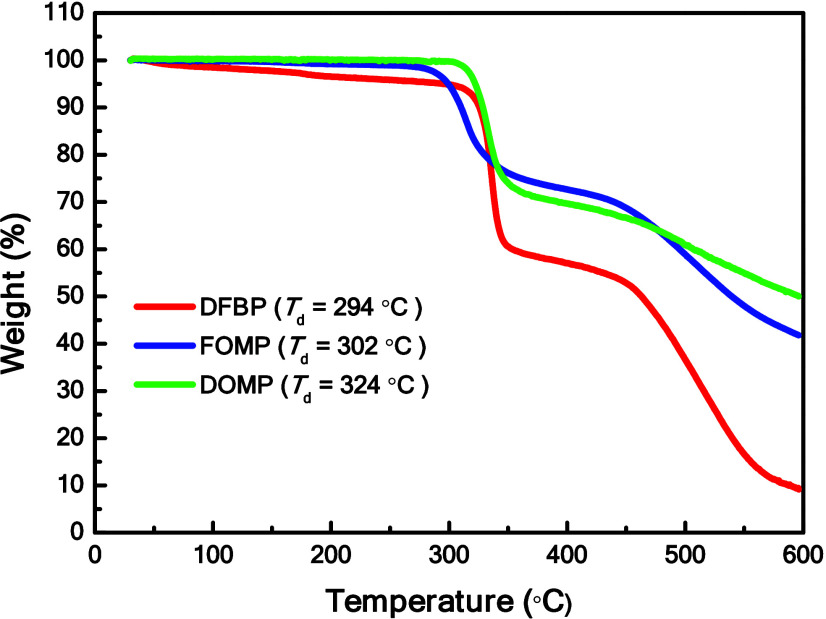
TGA curves of targeted complexes.

### Computational Analysis

The DFT calculations were performed at the B3LYP/(6–31G­(d,p)­LANL2DZ) level to gain deeper insights into their structures, optical properties, and electronic characteristics.^69^ The optimized S_0_ geometry and permanent dipole moments of the targeted complexes are shown in Figure S21. The calculated dipole moments of **DFBP**, **FOMP**, and **DOMP** are 10.94, 14.24, and 15.62 D, respectively. Notably, this trend is inversely correlated with their PLQY (Table 1). Such a trend supports the notion that smaller dipole moments facilitate higher PLQY, as the extent of triplet–triplet annihilation is closely associated with dipole magnitude.^70,71^ Figure 4 illustrates the calculated frontier molecular orbitals distribution. The HOMO is localized on the C^∧^N ligand and the central iridium metal, while the LUMO is distributed across the N^∧^N ligand and a small portion of the iridium metal. Such spatial separation between HOMO and LUMO suggests a potential MLCT character upon excitation.

**Figure 4:**
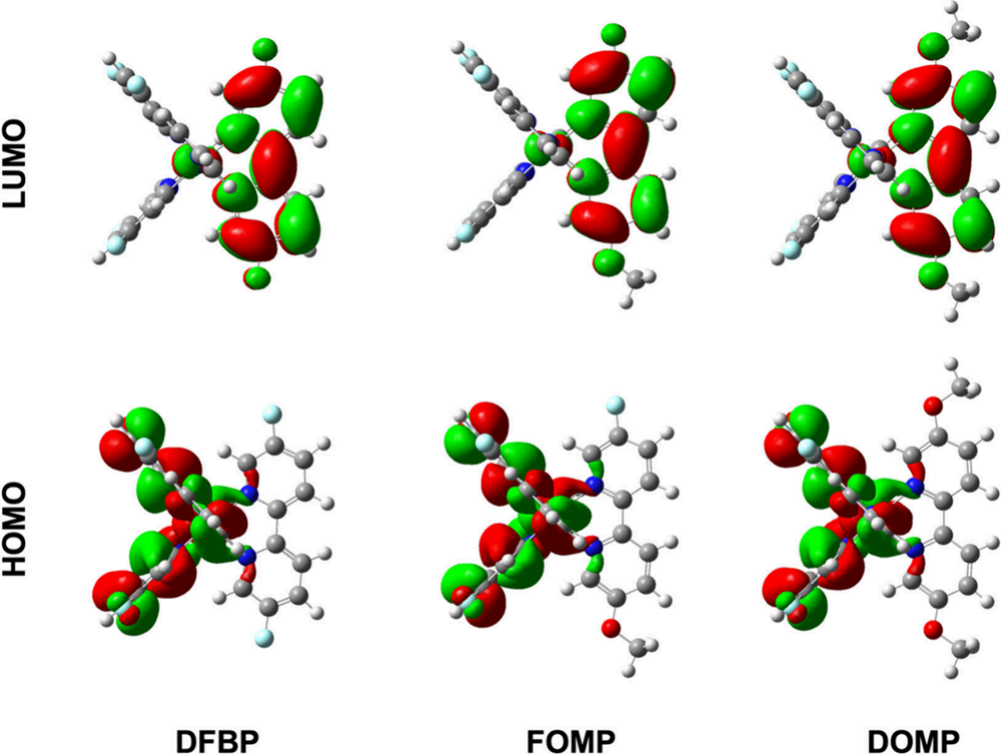
Schematic representation of energies calculated for the frontier molecular orbitals.

TD-DFT and NTOs analyses revealed configuration interaction coefficients and dominant orbital contributions, offering insights into the nature of the excited-state transitions.^72,73^ As summarized in Table S2, vertical excitations calculated at the optimized S_0_ geometries show distinct characteristics for each complex. For **DFBP**, the T_1_ state is primarily characterized by HOMO → LUMO transition (96.1%), indicative of a simple MLCT process and consistent with its observed featureless emission. In contrast, **FOMP** exhibits a mixed transition character. The T_1_ and T_2_ states are mainly associated with HOMO–5 → LUMO (27.4%) and HOMO → LUMO (71.6%) transitions, respectively. Notably, T_1_ and T_2_ states are close-lying in energy (0.01 eV), indicating near-degeneracy. To clarify the underlying nature of the transitions involved, NTOs analysis was performed (Figure 5(a)), revealing a combination of MLCT, LC, and a minor LLCT component. Accordingly, the T_1_ and T_2_ states are expected to collectively contribute to the emissive behavior of **FOMP**. In **DOMP**, TD-DFT calculations reveal that the T_1_ state primarily involves HOMO–2 → LUMO transition (80.0%) and exhibits LC character, whereas the T_2_ state is dominated by HOMO → LUMO transition (92.5%) and shows features of MLCT or LLCT. NTOs analysis reveals that both the hole and particle orbitals are predominantly localized on the same conjugated ligand, indicating a LC excited state with minimal metal involvement, which may be responsible for the reduced emission intensity observed experimentally.

**Figure 5:**
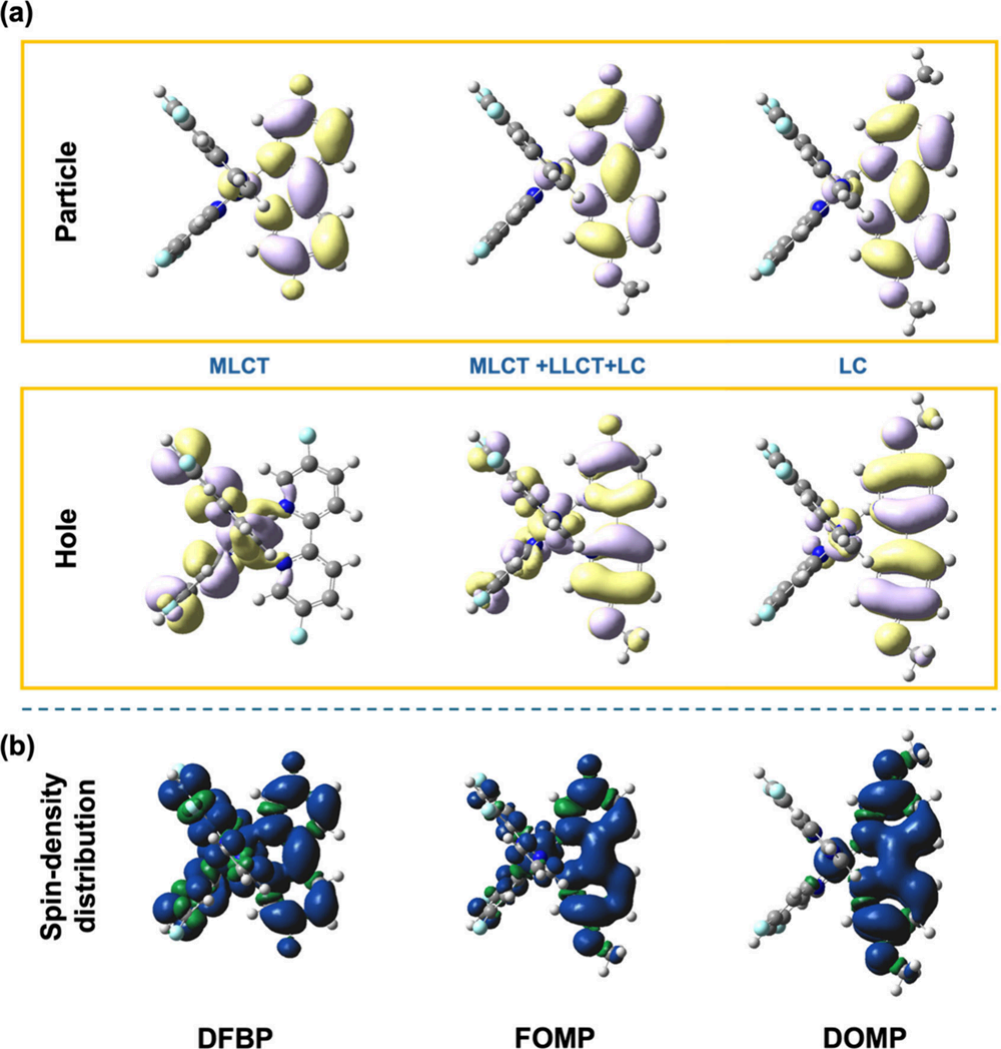
(a) Natural transition orbitals (S_0_ → T_1_) and (b) spin-density distributions in the triplet state for targeted complexes.

The geometries of the triplet state were optimized using spin-unrestricted DFT calculations at the UB3LYP level.^74–76^ In terms of structural changes, the selected bond lengths of S_0_ and T_1_ states are shown in Table S3. **DFBP** exhibits only minor variations upon excitation to the T_1_ state, indicating that the molecule retains a relatively rigid geometry in the excited state. In contrast, **FOMP** and **DOMP** undergo distortions, particularly in the Ir–N_(N^∧^N)_ bond lengths. Such relaxation may facilitate nonradiative deactivation pathways and ultimately compromise their photoluminescence performance. The spin-density distributions (Figure 5(b)) further support the electronic characteristics inferred from NTOs analysis. **DFBP** displays a delocalized spin density consistent with its MLCT nature, while **FOMP** shows more localized distribution onto the ligands, aligning with its mixed excited-state character (MLCT, LLCT, LC). However, in **DOMP**, the spin density is confined to the conjugated ligand, reinforcing its LC dominated configuration.

### Electroluminescent Characteristics of LECs

To evaluate the EL properties of the proposed complexes, LECs incorporating these complexes were fabricated and tested. The EL characteristics of these LECs are detailed in Table 3 The time-dependent EL spectra of LECs employing targeted complexes are shown in Figures S22–S24, respectively. The EL spectra of all complexes evolved with time because of the altered microcavity effect induced by the moving emission zone during device operation.^77,78^ The EL spectra were finally stabilized when the doping processes reached a steady state and the emission zone stopped moving. Thickness-dependent stabilized EL spectra of LECs employing targeted complexes are compared in Figure 6(a)–6(c), respectively. Microcaviy effect from different device thickness resulted in altered EL spectra from PL spectra, which suffered little microcaviy effect. In spite of microcaviy effect, the EL spectra of these complexes still resembled their PL spectra, implying similar PL and EL emission mechanisms.

**Figure 6:**
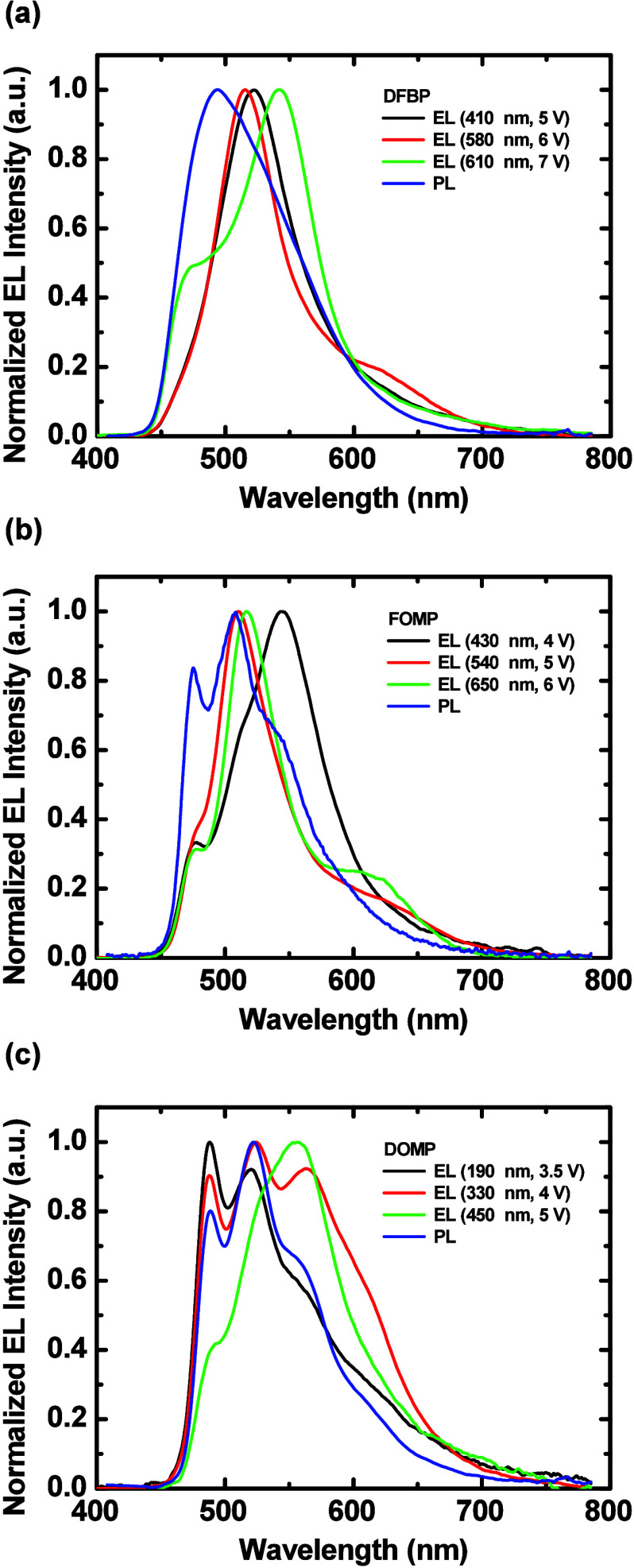
Thickness-dependent stabilized EL spectra of the LECs based on (a) **DFBP**, (b) **FOMP**, and (c) **DOMP**. The driving condition and thickness of each device are shown in the inset. The PL spectra of the emissive layers are also shown for comparison.

**Table 3:** Summary of the EL characteristics of the LECs based on the proposed complexes

Complex	Concentration (mg mL^–1^)^a^	Thickness (nm)	Operation voltage (V)	EL_max_ (nm)^b^	*B*_max_ (cd m^–2^)^c^	η_ext, max_ (%)^d^	η_C, max_ (cd A^–1^)^e^	η_P, max_ (lm W^1–^)^f^
**DFBP**	160	410	5	522	16.3	14.6	43.7	27.5
	200	580	6	516	22.5	16.8	47.2	24.7
	240	610	7	543	17.0	11.2	34.8	15.6
**FOMP**	160	430	4	543	9.3	12.1	43.6	34.2
	200	540	5	510	15.5	13.2	36.6	23.0
	200^g^	650	6	516	24.6	14.1	41.6	21.8
**DOMP**	80	190	3.5	488, 520, 565 (sh.), 628 (sh.)	8.3	4.3	11.9	10.7
	120	330	4	488, 524, 564, 615 (sh.)	25.7	7.3	21.5	16.9
	160	450	5	490, 527 (sh.), 556, 619 (sh.)	52.2	4.2	14.4	9.0

a Solution concentration for spin coating by 2000 rpm for 60 s.

b EL emission peak wavelength.

c Maximal brightness.

d Maximal external quantum efficiency.

e Current efficiency.

f Power efficiency.

g Spin coated at 1000 rpm. Others were spin coated at 1500 rpm.

The time-dependent current density, brightness, and EQE of the LECs based on **DFBP** with various emissive-layer thicknesses are shown in Figure 7(a)–7(c), respectively. After a constant bias was applied on the LEC, the mobile ions in its emissive layer moved toward electrodes, i.e., anions and cations toward anode and cathode, respectively, and thus the electrochemically doped layers were gradually formed. Such doped layers promoted carrier injection and enhanced the device current with time (Figure 7(a)). A higher bias voltage increased the electric field inside the device to fasten the ion redistribution, rendering a shorter device response time. As the device current increased, more excitons were generated and the brightness gradually enhanced as well (Figure 7(b)). However, the brightness decreased with time after reaching the peak value due to exciton quenching near the growing doped layers and material degradation.^79^ The device efficiency rapidly improved shortly after a bias was applied because the doped layers balanced electron and hole injection (Figure 7(c)). The EQE also decreased gradually after reaching the peak value, but the EQE decreased much earlier than brightness. It further confirmed that exciton quenching near the extending doped layers was significant during the whole device operation time. As such, the EQE started to deteriorate while the brightness was still increasing. The LECs employing **FOMP** and **DOMP** also showed similar temporal EL characteristics (Figure S26 and Figure S27).

**Figure 7:**
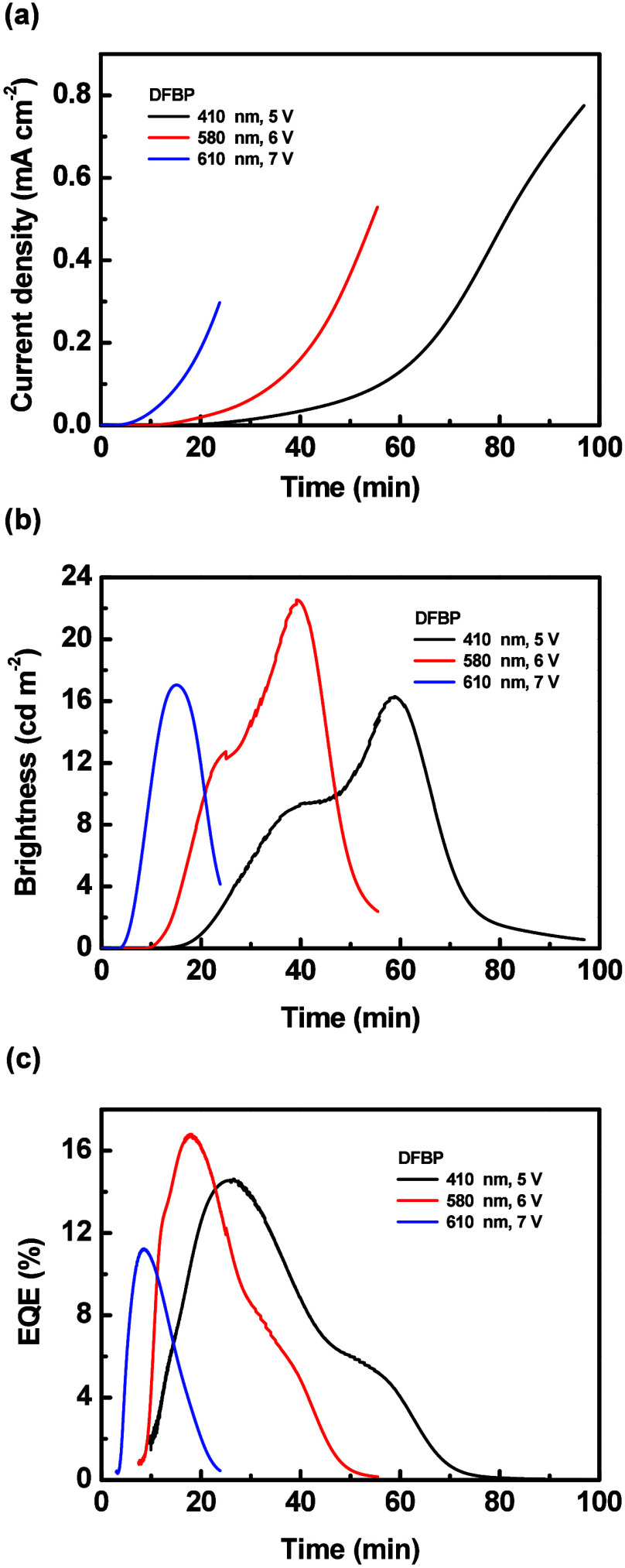
Time-dependent (a) current density, (b) brightness, and (c) EQE of the LECs based on **DFBP**. The driving condition and thickness of each device are shown in the inset.

The peak EQE of the optimized LECs based on **DFBP** (580 nm), **FOMP** (650 nm), and **DOMP** (330 nm) reached 16.8, 14.1 and 7.3%, respectively. Most importantly, as depicted in Figure 8, the peak EQEs obtained from the LECs employing **DFBP** and **FOMP** are among the highest reported values in blue-green LECs.^38^ This highlights the significant potential of these materials for advancing LEC technology. However, compared to high EQEs (ca. 25–35%) achieved in the reported blue phosphorescent OLEDs based on efficient iridium complexes,^80–83^ the EQEs of the LECs employing the proposed complexes were still not sufficiently high. Further improving the device efficiency would be necessary.

**Figure 8:**
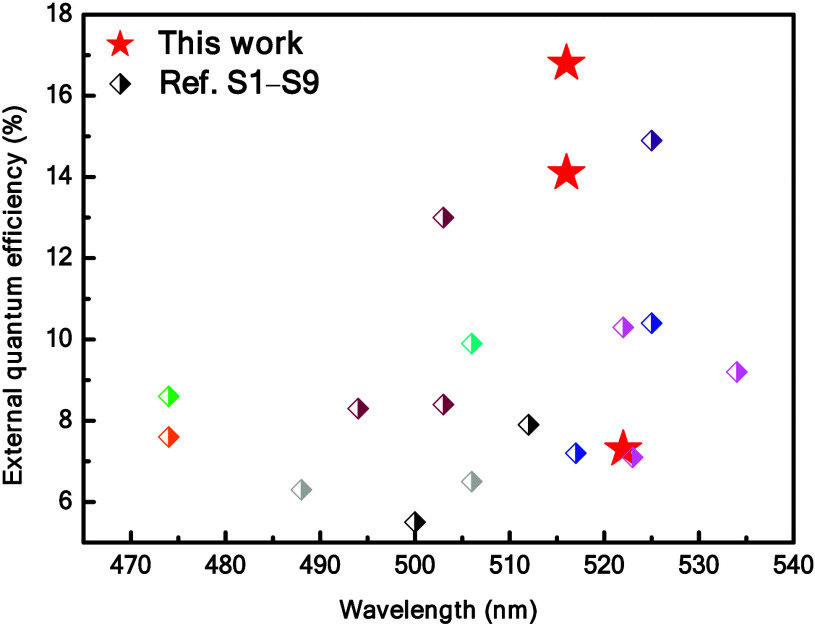
Summary of EQE > 5% for representative blue-green LECs with emission peaks between 470 and 535 nm.

The measured EQE of the LECs were highly correlated with the PLQY of their emissive layers (Table 1). The eq (eq 1) shown below considers the parameters that determine the EQE of LECs based on these complexes.

1 $$\eta_{EQE}=\eta_{out}\times \gamma \times \eta_{S,T}\times \eta_{QY}$$

In this equation, η_EQE_ is the measured device EQE, η_out_ is the optical outcoupling efficiency, γ is the factor of carrier balance in the LEC, η_S,T_ is the emissive exciton generation efficiency of the complex, and η_QY_ is the thin-film PLQY of the complex. For phosphorescent complexes, both singlet and triplet excitons can be harvested (η_S,T_ = 100%). When estimating the optical outcoupling efficiency (ca. 20–30%) of an LEC device^58,84^ and the PLQY of the emissive layers (Table 1), the estimated factors of carrier balance (γ) of the LECs based on **DFBP**, **FOMP**, and **DOMP** are close to the perfect values (100%). These data reveal that the proposed complexes exhibit superior carrier balance when employed in LECs. However, the ancillary ligands show significant impact on the PLQY of the complexes. It may be attributed to the fact that the methoxy-substituted ancillary ligands exhibit decreased rigidity and thus lower PLQY. The fluorinated-substituted ancillary ligand is beneficial in increasing the rigidity and improves the PLQY.

It is interesting to compare the carrier balance of the LECs based on the iridium complexes with different ancillary ligands. We have previously reported an iridium complex with two C^∧^N ligands (**dfppz**) and an ancillary ligand (4,4′-dimethyl-2,2′-bipyridine, **dmbpy**).^21^ It showed a high PLQY of 93%^58^ and a good device EQE of 14%. However, the estimated factor of carrier balance (γ) of the optimized LEC based on complex [Ir­(dfppz)_2_(dmbpy)]­PF_6_ was only ca. 76%. It reveals that the complexes with ancillary ligands containing high-polarity substituents exhibit better carrier balance when employed in LECs. Among the proposed ligands, the fluorinated-substituted ancillary ligand is preferred to achieve good carrier balance and high PLQY simultaneously. Therefore, a higher EQE can be obtained from the LEC based on **DFBP**, which shows a lower PLQY than complex [Ir­(dfppz)_2_(dmbpy)]­PF_6_.

To further enhance the device efficiency, recycling the light trapped in substrate and waveguide modes is an effective approach.^85–88^ It can be achieved by inserting a diffusive layer composed of a TPR layer doped with TiO_2_ nanoparticles between ITO layer and glass substrate.^21,57,58,84,89,90^ The optimized LECs based on **DFBP** (580 nm) and **FOMP** (650 nm), which showed better device efficiencies, were chosen to be integrated with the diffusive substrates for further enhanced light extraction and their EL characteristics are summarized in Table 4 and Table S4, respectively. The time-dependent EL spectra of the optimized LECs based on **DFBP** (580 nm) integrated with the diffusive substrates under 3.5, 5, and 7 V are shown in Figure 9(a)–9(c), respectively. The diffusive substrate reduced the microcavity effect due to the scattered optical feedback from the substrate reflection. Therefore, compared with the EL spectra from the LECs without diffusive substrates (Figure S23­(b)), less altered EL spectra, i.e., more similar to the PL spectra, can be obtained when the LECs were integrated with the diffusive substrates. Similarly, the optimized LECs based on **FOMP** (650 nm) integrated with the diffusive substrates also showed less altered EL spectra (cf. Figure S24­(c) and Figure S28). These data reveal that the diffusive substrates are beneficial in mitigating the microcavity effect and recovering the intrinsic EL spectra of the LECs.

**Figure 9:**
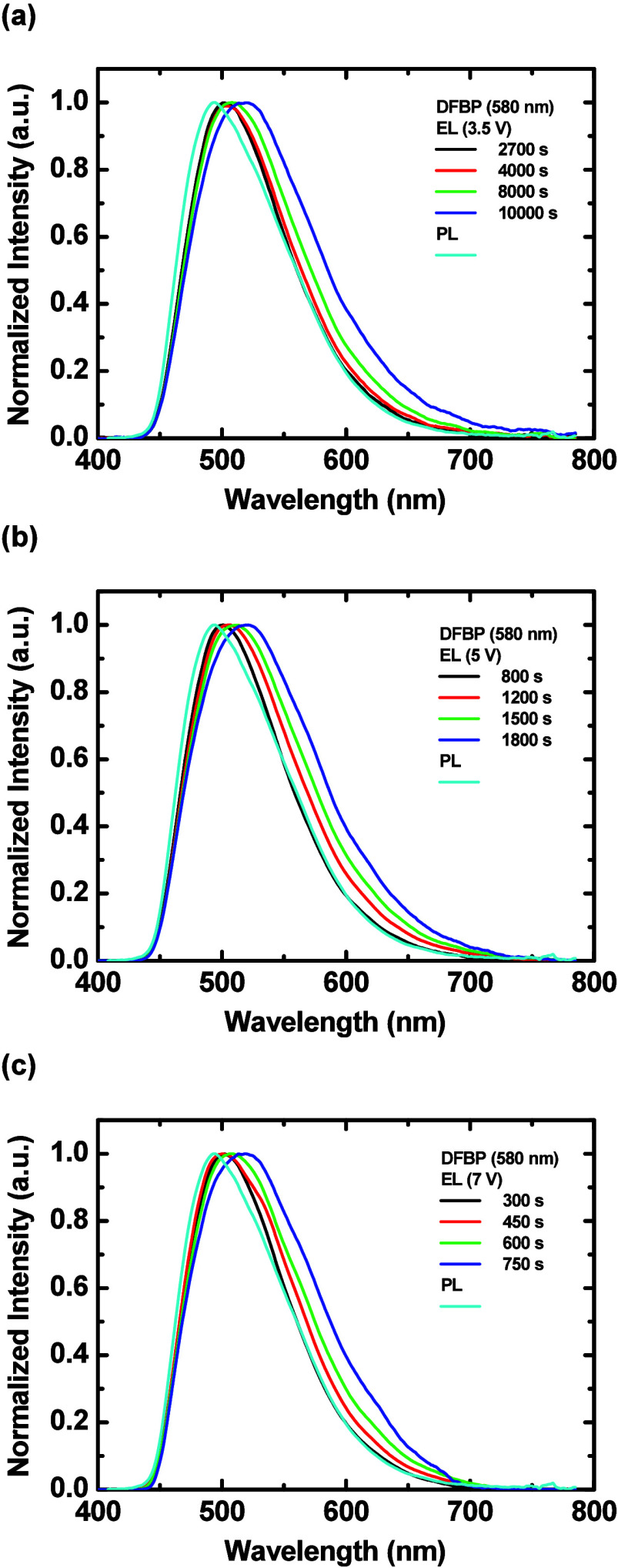
Time-dependent EL spectra of the optimized LECs based on **DFBP** (580 nm) integrated with the diffusive substrates under (a) 3.5, (b) 5, and (c) 7 V.

**Table 4:** Comparison of the EL characteristics of the optimized LECs based on DFBP (580 nm) without and with diffusive substrates

Device	Operation voltage (V)	E*L*_max_ (nm)^c^	*B*_max_ (cd m^–2^)^d^	η_ext, max_ (%)^e^	η_C, max_ (cd A^–1^)^f^	η_P, max_ (lm W^1–^)^g^
Without diffusive substrate^a^	3.5	497	2.0	14.5	40.2	36.1
	5	504	13.7	12.2	33.1	20.8
	7	496	60.6	9.3	25.4	11.4
With diffusive substrate^b^	3.5	519	4.9	39.3	109.6	98.3
	5	519	43.1	32.3	89.9	56.5
	7	518	127.8	25.3	71.1	31.9

a LECs fabricated on ITO (160 nm)/glass substrates.

b LECs fabricated on ITO (160 nm)/diffusive layer/glass substrates.

c EL emission peak wavelength.

d Maximal brightness.

e Maximal external quantum efficiency.

f Current efficiency.

g Power efficiency.

In addition to reduced spectral alternation, the diffusive layer redirected some of the trapped light in the glass substrate and ITO layer into the forward direction, significantly enhancing the light output. The time-dependent EL characteristics of the optimized LECs based on **DFBP** (580 nm) and **FOMP** (650 nm) integrated with the diffusive substrates are depicted in Figure 10 and Figure S29, respectively. These EL properties were similar to those obtained from the LECs without the diffusive substrates (cf. Figure 7 and Figure S26). However, with the diffusive substrates, the peak EQEs (current efficiencies) of the optimized LECs based on **DFBP** (580 nm) and **FOMP** (650 nm) were enhanced to 39.3% (109.6 cd A^–1^) and 35.6% (98.3 cd A^–1^), respectively. For comparison, the reference device (without diffuser) data of the optimized LECs based on **DFBP** (580 nm) and **FOMP** (650 nm) fabricated on the ITO layers with the same thickness of that on the diffusive substrates (160 nm) are also included in Table 4 and Table S4, respectively. The time-dependent EL spectra and the time-dependent EL characteristics of the reference LECs based on **DFBP** (580 nm) are shown in Figure S30 and Figure S31, respectively. The time-dependent EL spectra and the time-dependent EL characteristics of the reference LECs based on **FOMP** (650 nm) are shown in Figure S32 and Figure S33, respectively. With the diffusive substrates, the device efficiencies of the LECs based on **DFBP** and **FOMP** have been enhanced by 269 and 274%, respectively, in comparison with their reference LECs. The diffusive substrates indeed significantly improved the light extraction from the LECs. These record-high device efficiencies indicate that highly efficient LECs can be achieved by employing well-designed iTMCs with superior carrier balance and outstanding light extraction techniques.

**Figure 10:**
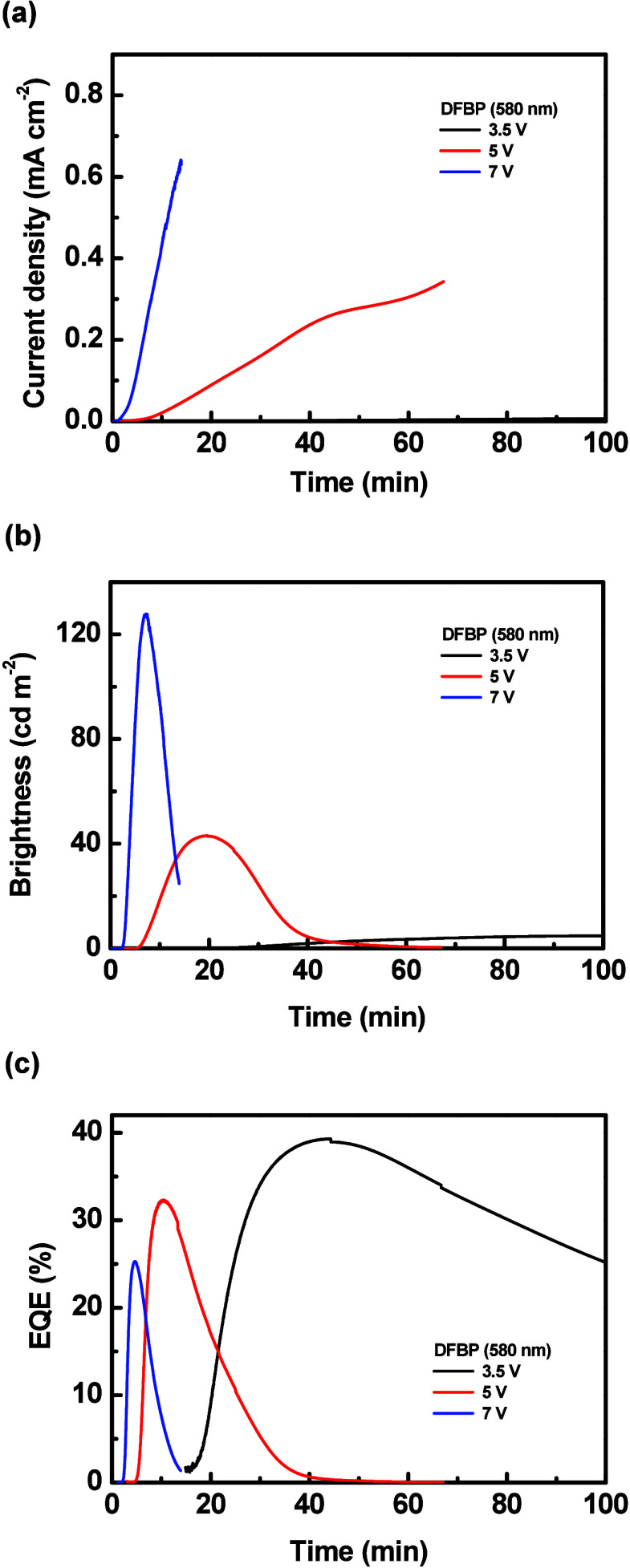
Time-dependent (a) current density, (b) brightness, and (c) EQE of the optimized LECs based on **DFBP** (580 nm) integrated with the diffusive substrates under 3.5, 5, and 7 V. The driving condition of each device is shown in the inset.

## Conclusions

A series of high-efficiency blue-green emitting cationic Ir­(III) complexes have been successfully designed and synthesized for application in LECs. This study indicates that F-substituents have a significant impact on emission performance, including the enhancement of PLQY and electroluminescent efficiency observed in LECs. When utilized as the emitting layer in LECs, **DFBP** exhibited the highest luminous efficiency in this series, achieving an EQE of 16.8% and current efficiency of 47.2 cd A^–1^. To the best of our knowledge, this targeted complex represents the record-high EQE reported for blue-green LECs. Furthermore, by integrating a diffusive layer to enhance light extraction, the EQE was boosted by ca. 270%, reaching 39.3%, while the current efficiency increased to 109.6 cd A^–1^. This molecular design strategy significantly improves the efficiency of LECs, providing new directions and potential for the development of next-generation cost-effective and large-area lighting technologies.

## Supplementary Material



## Data Availability

Data will be made available on request.

## Notes

^1^H, ^13^C, DEPT-90 NMR spectra, high resolution mass spectra, PL spectra, transient PL curves, thermal analysis, theoretical calculation, and EL spectra (PDF)
