# Supplementary material for: Record-High Efficiency Blue-Green Cationic Ir(III) Complexes for Light-Emitting Electrochemical Cells with EQE Approaching 40%
Source: Inorg Chem. 2025 May 19;64(21):10402–13. doi: 10.1021/acs.inorgchem.5c00167 (PMC12135039; doi:10.1021/acs.inorgchem.5c00167)
Supplement: Supplementary file 1 [file ic5c00167_si_001.pdf]

Supporting Information

Record-high Efficiency Blue-green Cationic Ir(III) Complexes for Light-emitting Electrochemical Cells with EQE Approaching 40%

Yu-Ting Huang,[a+] Chung-Chieh Chang,[b+] Che-Lun Chang,[a] Wei-Tse Hsu,[c] Yun-Rong Li,[a] Zu-Po Yang,[c] Chin-Wei Lu,*[a] and Hai-Ching Su*[b]

[a] Department of Applied Chemistry, Providence University, Taichung 43301, Taiwan

[b] Institute of Lighting and Energy Photonics, National Yang Ming Chiao Tung University, Tainan 71150, Taiwan

[c] Institute of Photonic System, National Yang Ming Chiao Tung University, Tainan 71150, Taiwan

[+] Equal contribution

* Corresponding authors

E-mail: cwlu@pu.edu.tw ; Fax: +886-4-26327554; Tel: +886-4-26328001-15213 (C.-W. Lu)

E-mail: haichingsu@nycu.edu.tw ; Fax: +886-6-3032535; Tel: +886-6-3032121-57792 (H.-C. Su)

Table of Contents

General information 3

^{1}H, ^{13}C, DEPT-90 NMR spectra 5

Mass spectra 10

PL spectra 11

Transient PL curves 13

Thermal analysis 17

Theoretical calculation 18

EL spectra 23

Reference 36

General information

Synthesis and Characterization. Unless otherwise specified, all commercial reagents were purchased and used as received without further purification. ^{1}H NMR spectra were recorded at 400 MHz, and ^{13}C/DEPT-90 NMR spectra were recorded at 100 MHz NMR instruments in CD_3CN solutions and chemical shifts were referenced to residual solvent. If CD_3CN was used as solvent, ^{1}H and ^{13}C NMR spectra were recorded with CD_3CN (^{1}H: δ = 1.94 ppm; ^{13}C: δ = 118.26 ppm) as internal references, respectively. The following abbreviations (or combinations thereof) were used to explain ^{1}H NMR multiplicities: s = singlet, d = doublet, t = triplet, q = quartet, p = quintet, m = multiplet.

Electrochemistry. Cyclic voltammetry was performed using a CHI 611E electrochemical analyzer according. 0.1 M tetra-n-butylammonium hexafluorophosphate was used as the supporting electrolyte, anhydrous MeCN, was used as the solvents for the E_{ox} and E_{red} measurements, and the solutions were bubbled with nitrogen for 10 min prior to the test. Silver wire, platinum wire and glassy carbon were used as pseudo reference electrode, counter electrode, and working electrode respectively. Scan rate was 100 mV s^{-1}. The redox potentials are based on the values measured from different pulsed voltammetry and are reported relative to an internal reference ferrocenium/ferrocene (Cp_2Fe/Cp_2Fe^{+}).

Computational details. The theoretical calculations of the Ir(III) complexes were performed using Gaussian 09. The molecular geometries of ground states (S_0) were optimized with the density functional theory (DFT) method. The TD-DFT

calculations were performed using a B3LYP function with a basis set of 6-31G(d,p)[F,O,N,C,H] and a LANL2DZ basis set for Ir atom. Natural transition orbital (NTO) analysis was employed to provide a clear and compact representation of electronic excitations involving multiple orbital transitions without a single dominant contribution, such as the $S_0 \rightarrow T_1$ excitation examined at the optimized S_0 structure. The T_1-state geometry was optimized using the spin-unrestricted UB3LYP method with a triplet spin multiplicity.

Photophysical Measurements. Photophysical characteristics of complexes in solutions were collected at room temperature using 1×10^{-5} M MeCN solutions of all complexes on spectrofluorometer Edinburgh FS5, which were carefully purged with nitrogen prior to measurements. UV-Vis absorption spectra were recorded on Perkin Elmer Lambda 14 spectrophotometer.

^1H, ^{13}C, DEPT-90 NMR Spectra

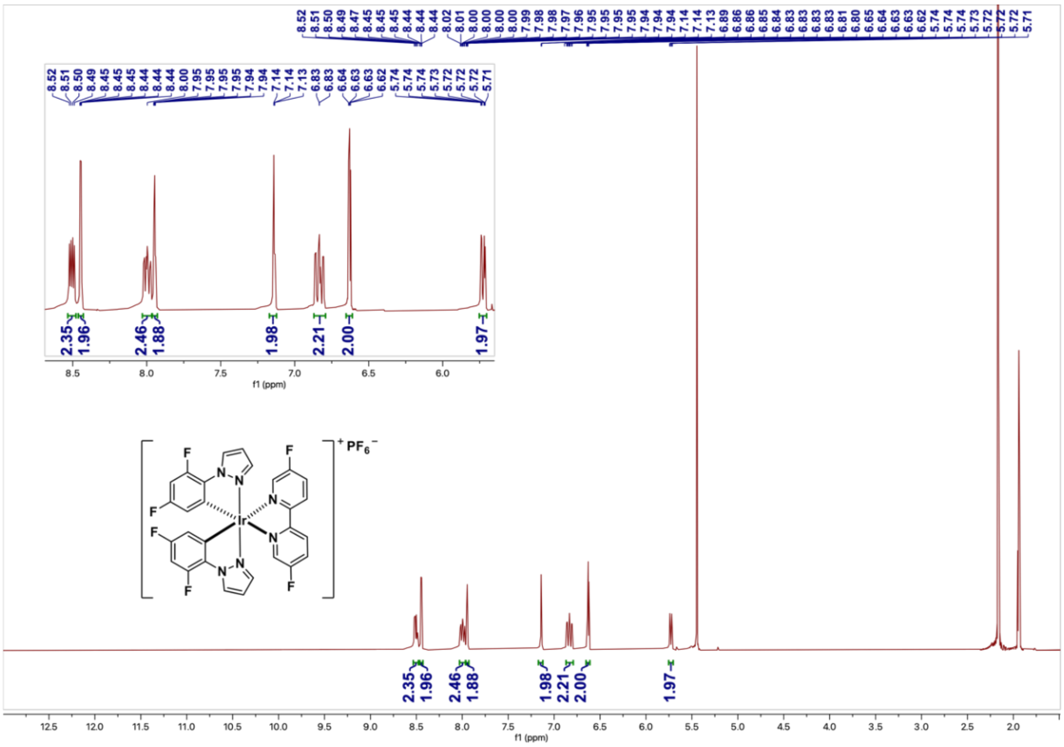

Figure S1. ^1H spectrum of **DFBP** (400 MHz, CD_3CN, 298 K).

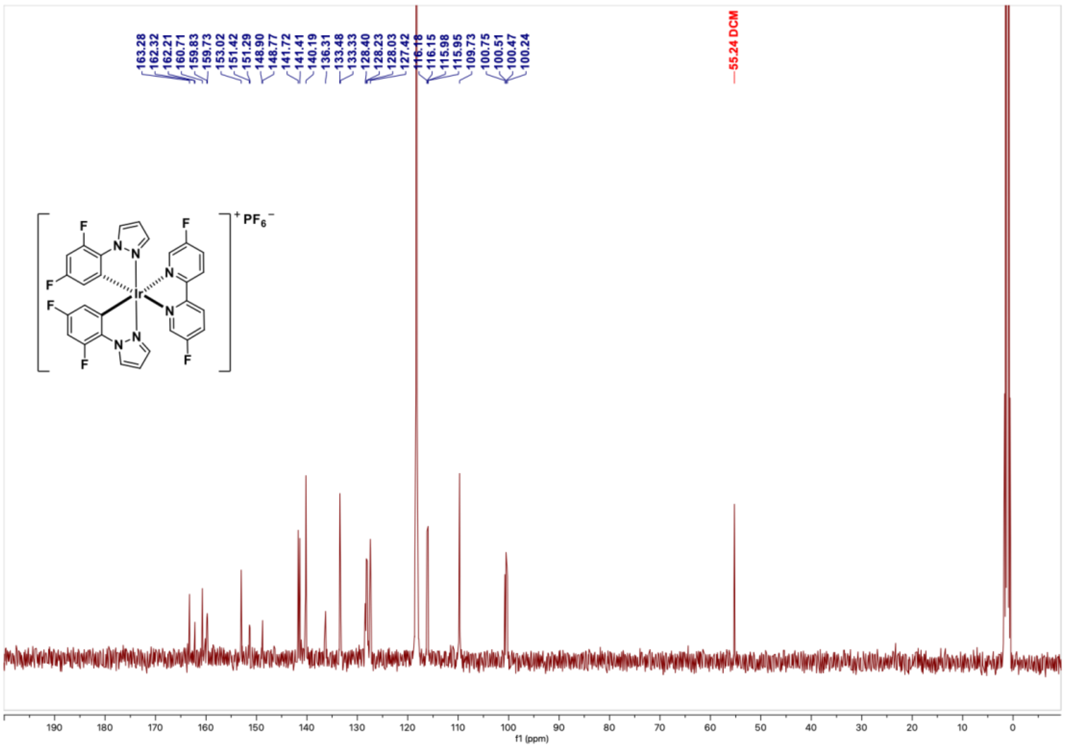

Figure S2. ^{13}C NMR spectrum of **DFBP** (100 MHz, CD_3CN, 298K).

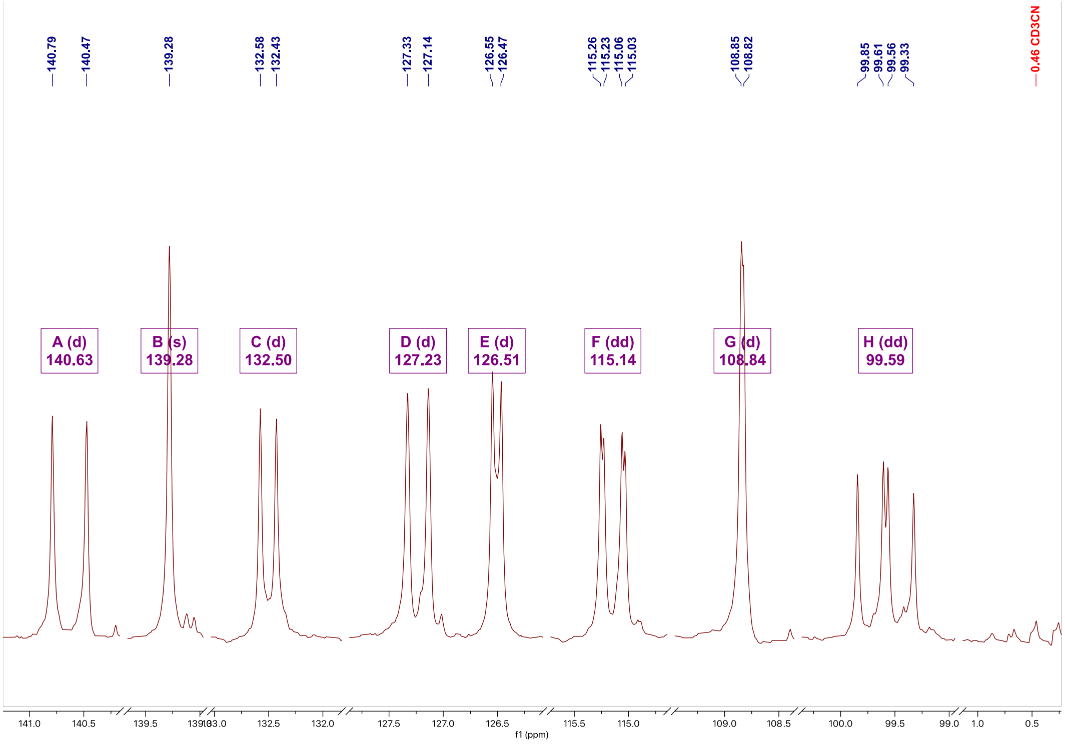

Figure S3. DEPT-90 NMR spectrum of **DFBP** (100 MHz, CD_3CN, 298K).

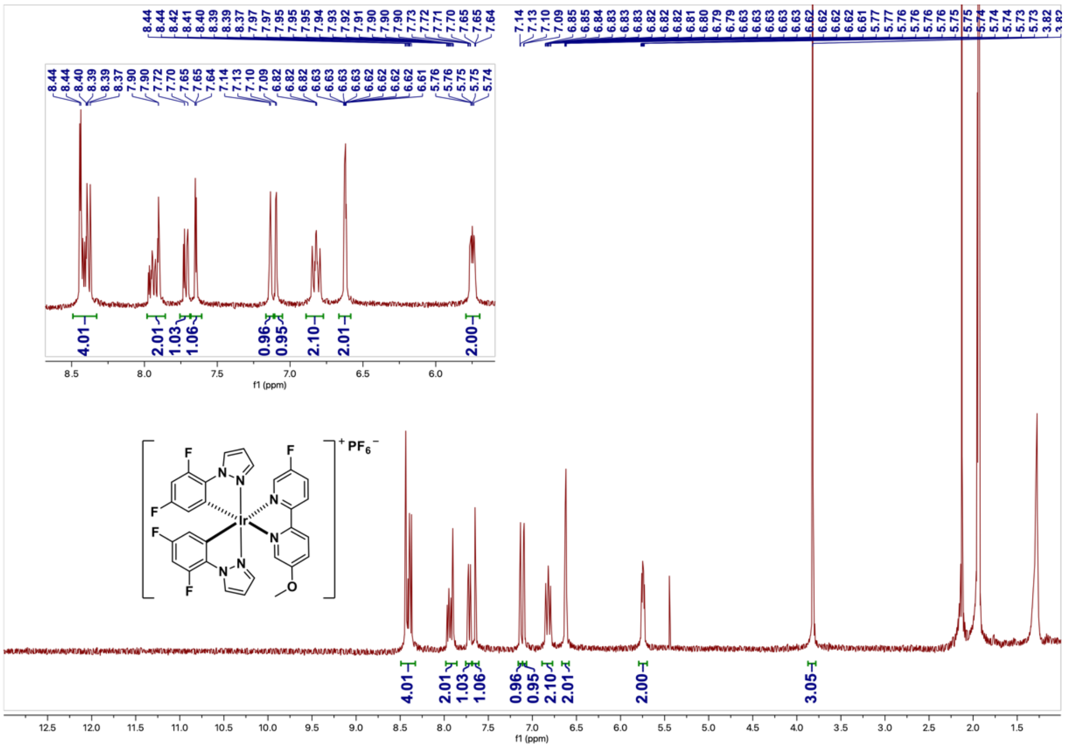

Figure S4. ^1H NMR spectrum of **FOMP** (400 MHz, CD_3CN, 298K).

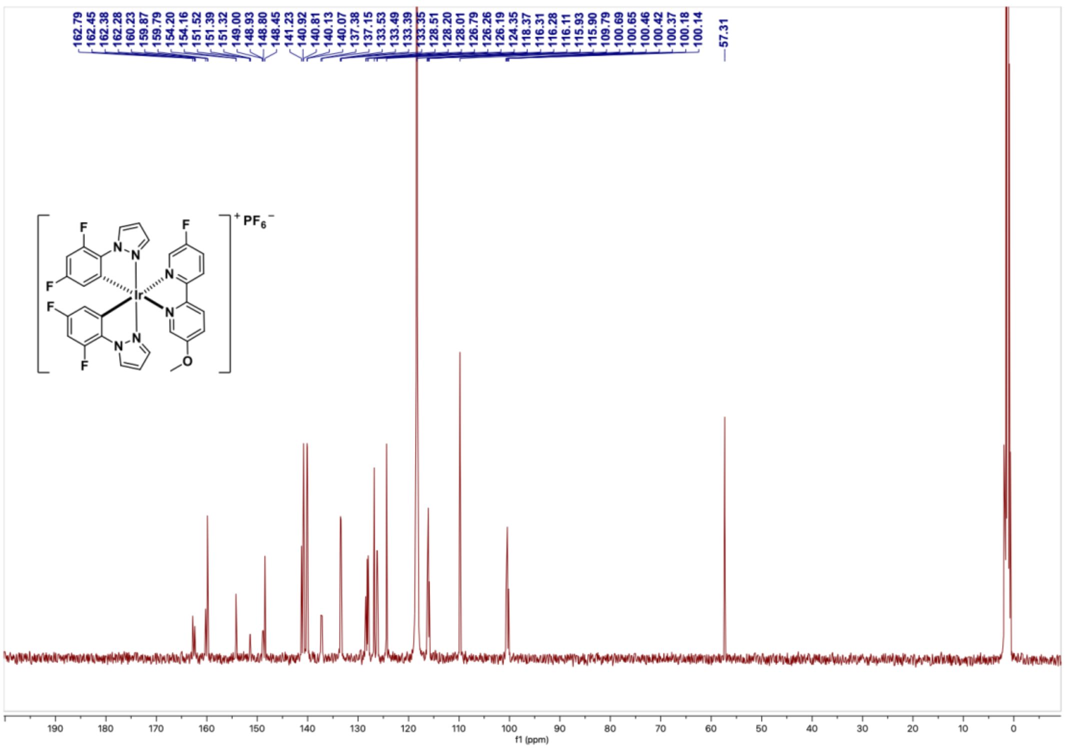

Figure S5. ^{13}C NMR spectrum of **FOMP** (100 MHz, CD_3CN, 298K).

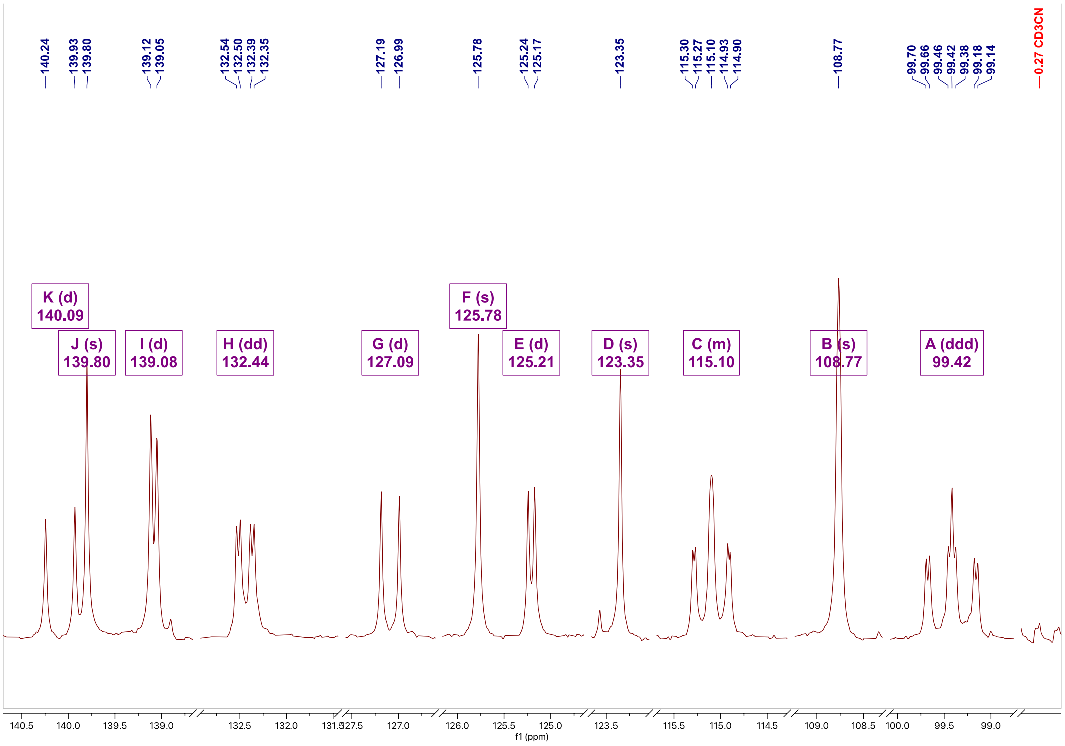

Figure S6. DEPT-90 NMR spectrum of **FOMP** (100 MHz, CD_3CN, 298K).

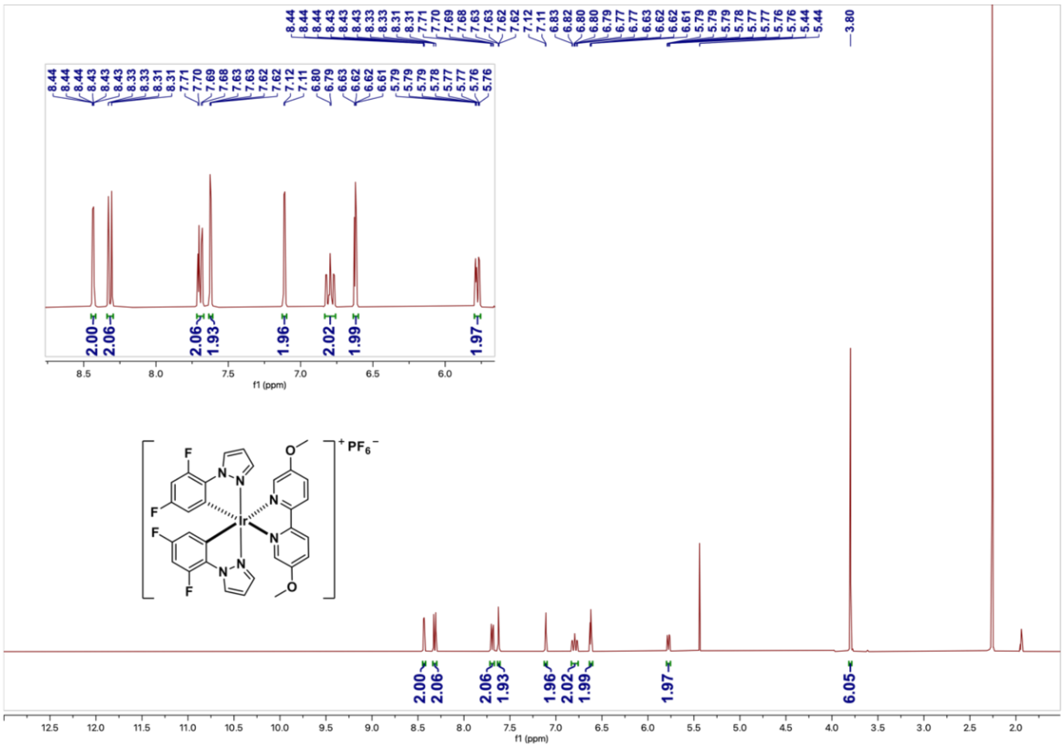

Figure S7. ^{1}H NMR spectrum of **DOMP** (400 MHz, CD_3CN, 298K).

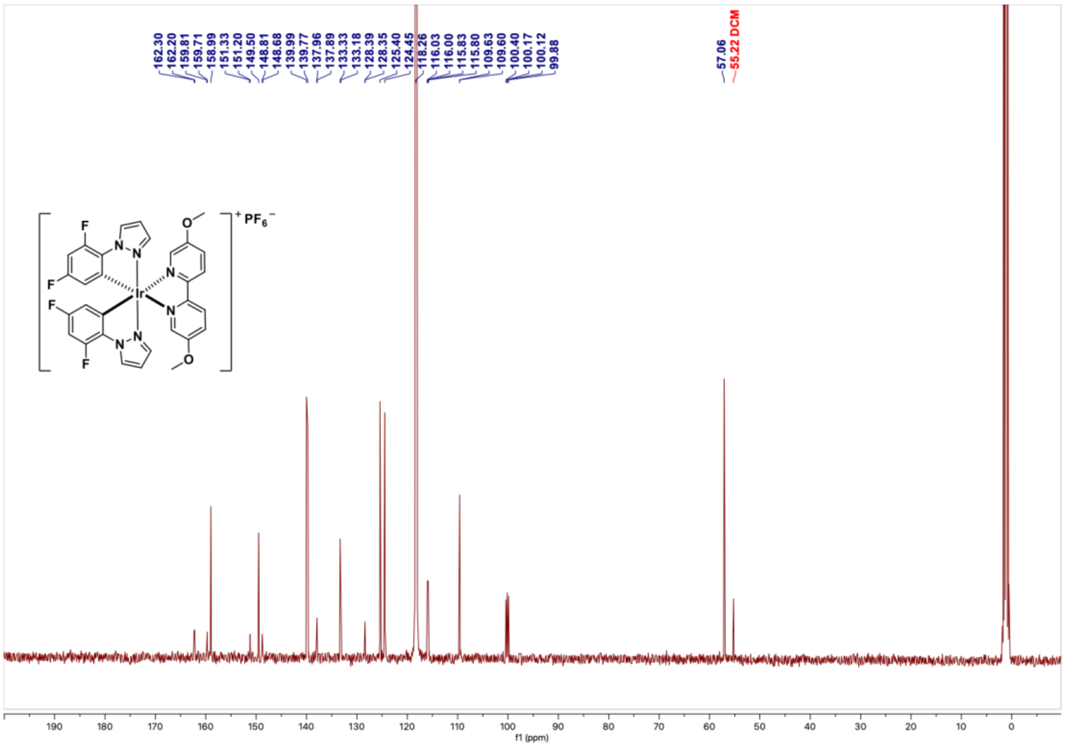

Figure S8. ^{13}C NMR spectrum of **DOMP** (100 MHz, CD_3CN, 298K).

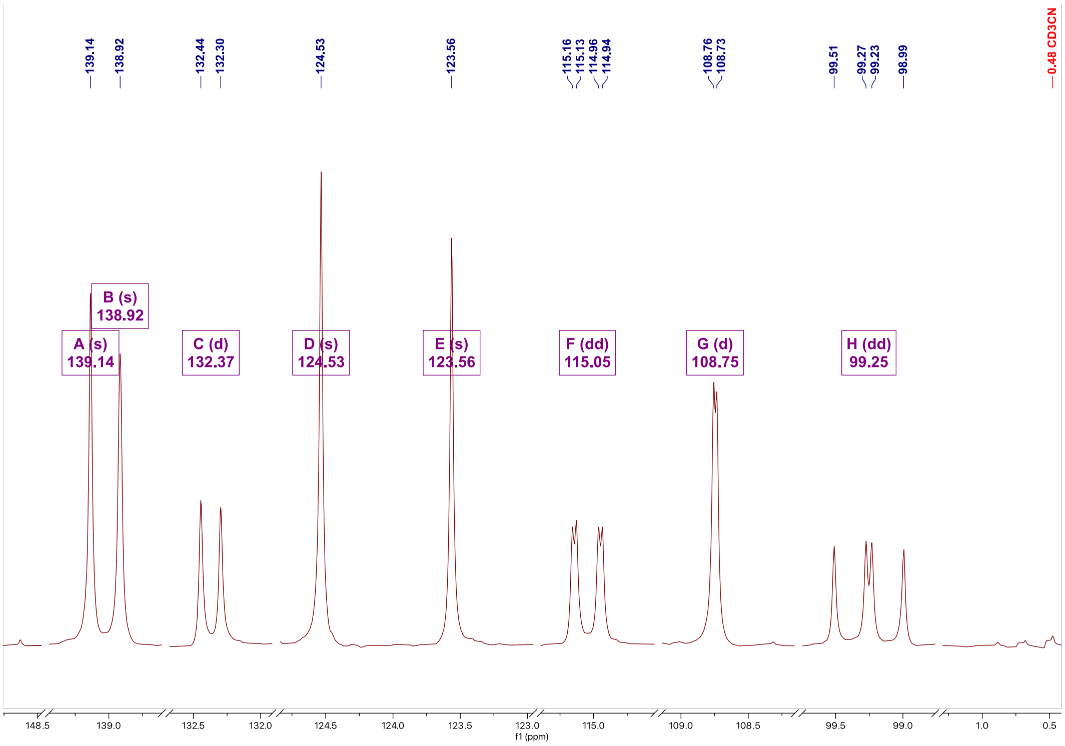

Figure S9. DEPT-90 NMR spectrum of **DOMP** (100 MHz, CD_3CN, 298K).

Mass Spectra

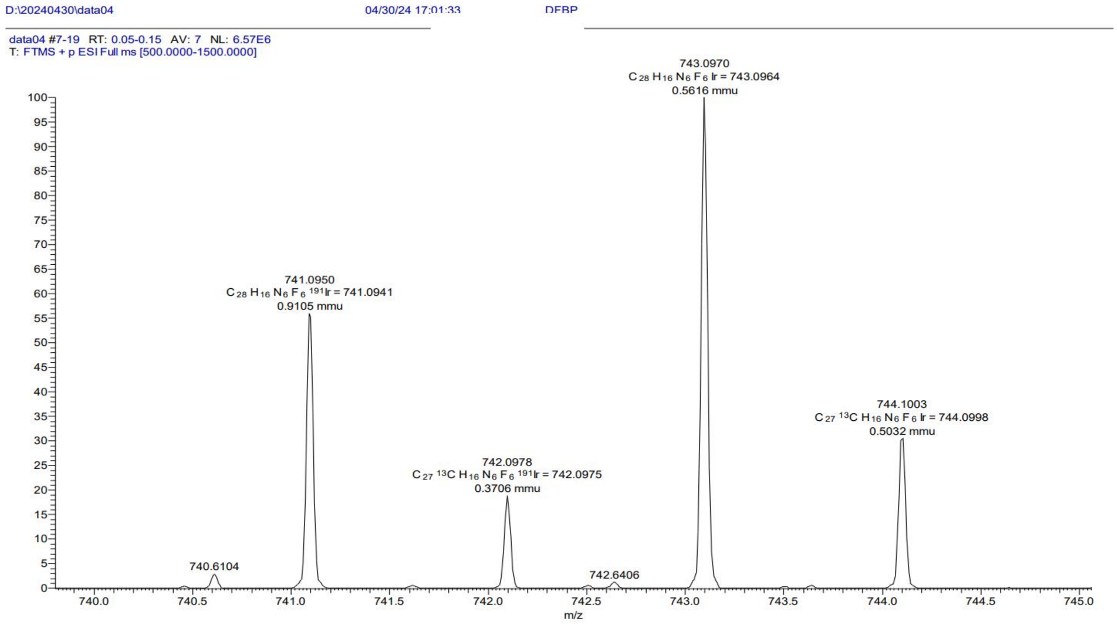

Figure S10. HRMS-ESI^{+} spectrum of **DFBP**.

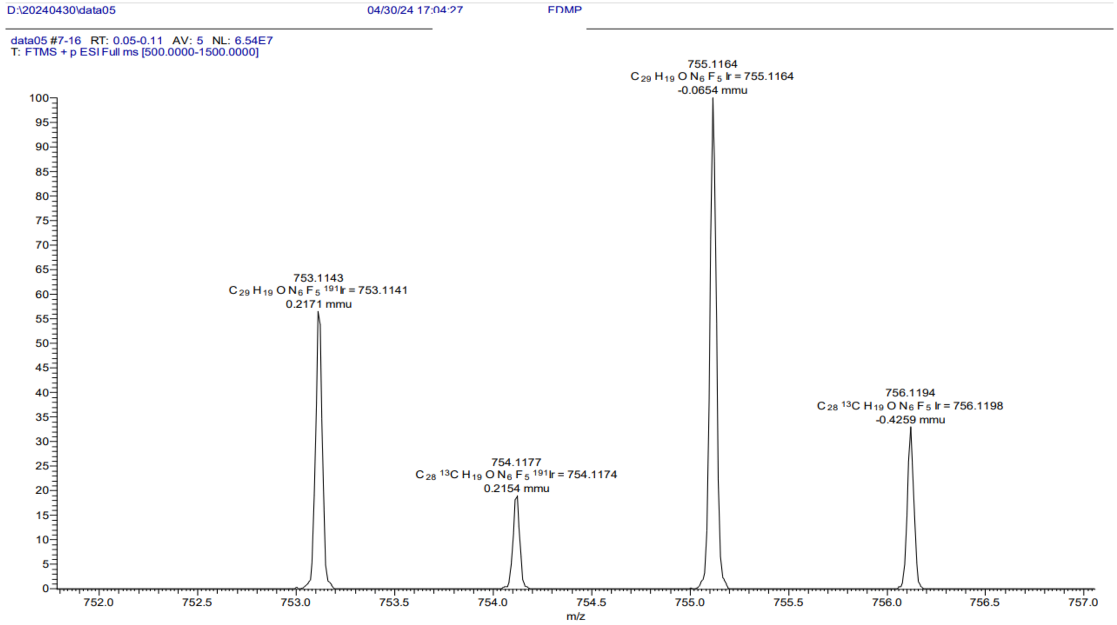

Figure S11. HRMS-ESI^{+} spectrum of **FOMP**.

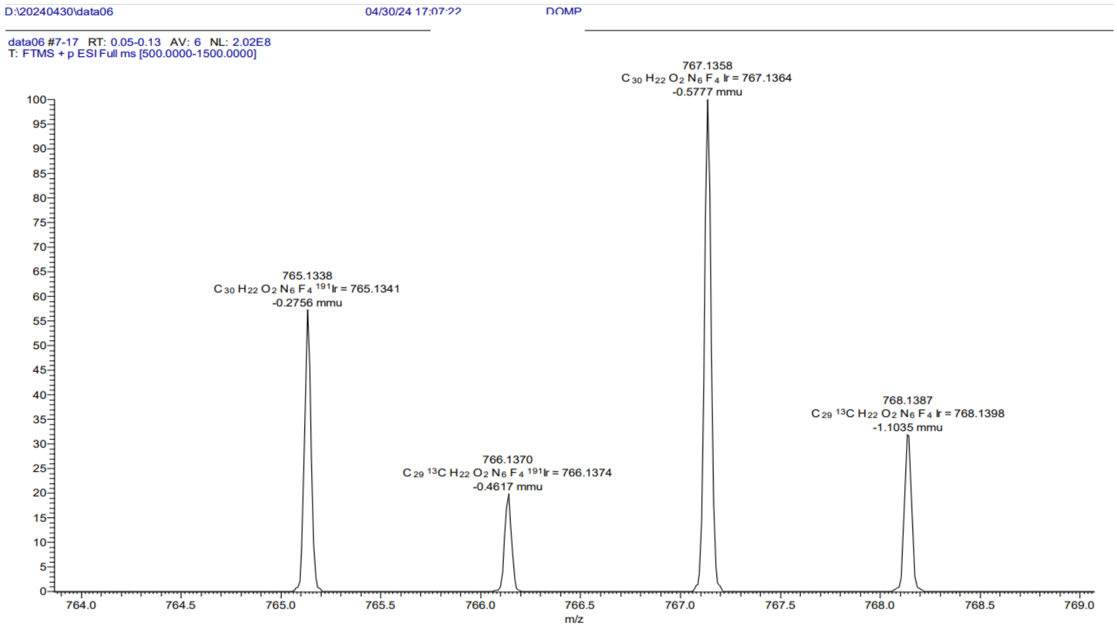

Figure S12. HRMS-ESI$^+$ spectrum of **DOMP**.

PL Spectra

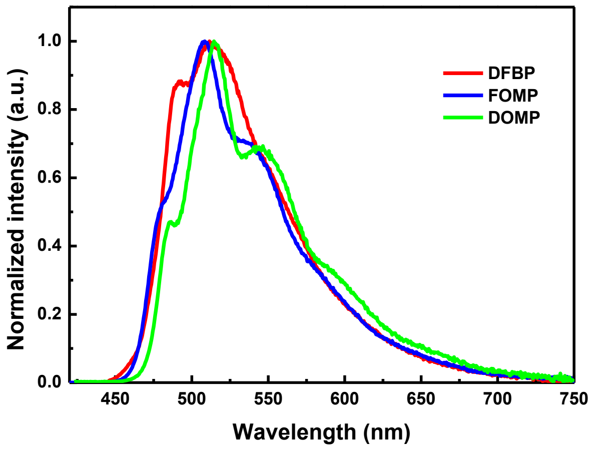

Figure S13. PL spectra of **DFBP**, **FOMP**, and **DOMP** in powder at 298 K after excitation at 320 nm.

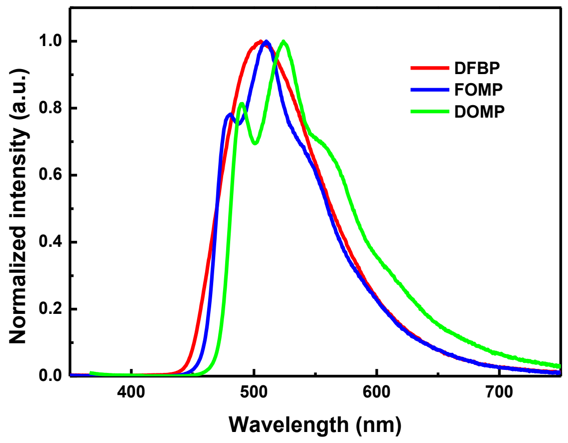

Figure S14. PL spectra of **DFBP**, **FOMP**, and **DOMP** in neat film at 298 K after excitation at 320 nm.

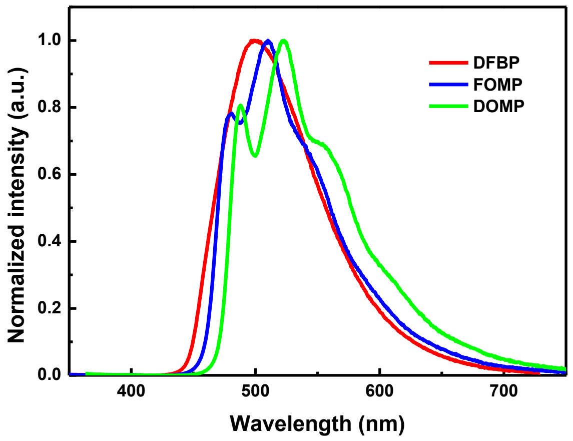

Figure S15. PL spectra of **DFBP**, **FOMP**, and **DOMP** in the thin-film contained complex (80 wt.%) and [BMIM$^+$(PF$_6^-$)] (20 wt.%) at 298 K after excitation at 320 nm.

Transient PL Curves

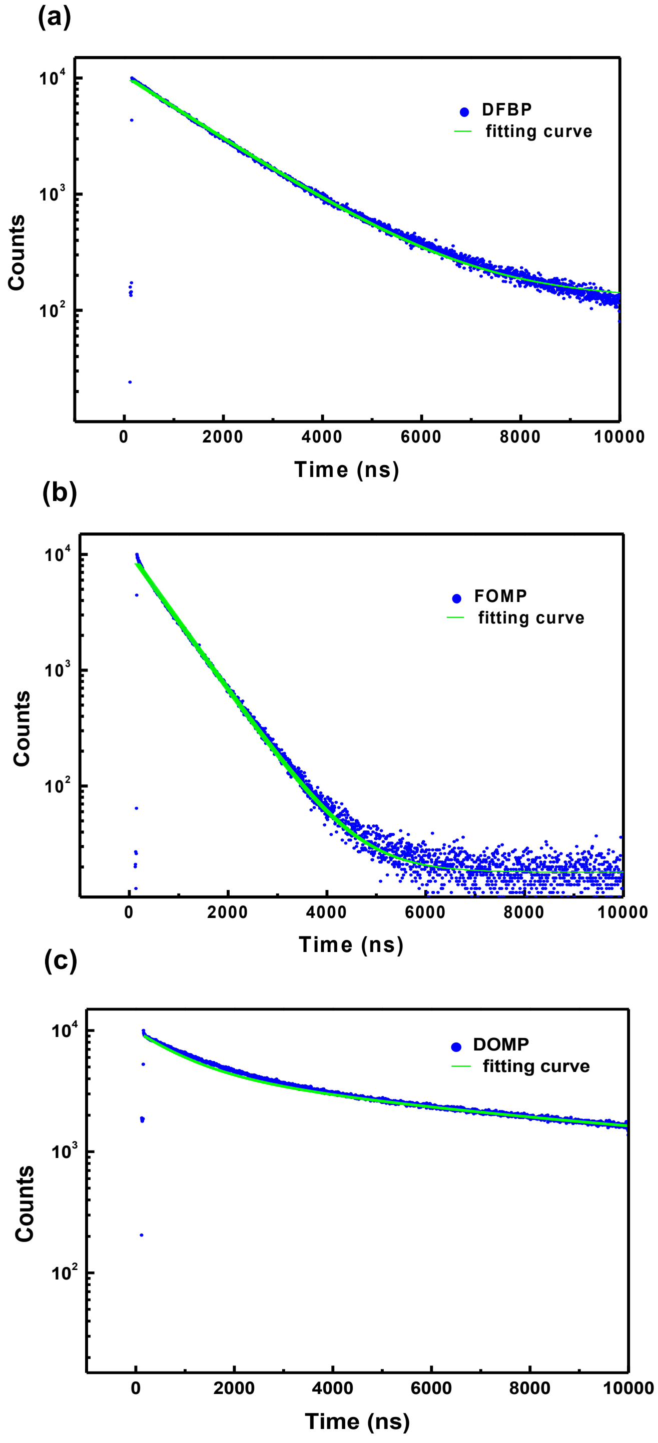

Figure S16. TrPL decay and fitting curve of (a) **DFBP**, (b) **FOMP**, and (c) **DOMP** in acetonitrile solution at 298 K after excitation at 320 nm.

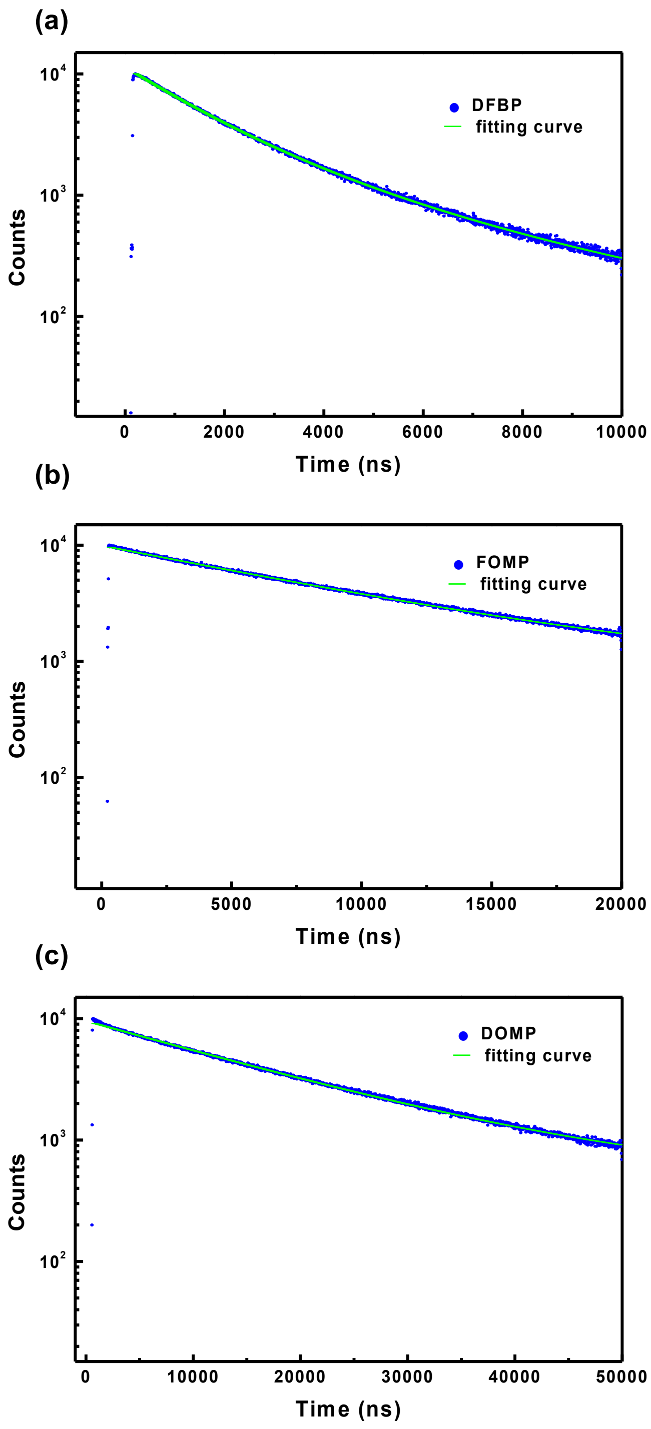

Figure S17. TrPL decay and fitting curve of (a) **DFBP**, (b) **FOMP**, and (c) **DOMP** in powder at 298 K after excitation at 320 nm.

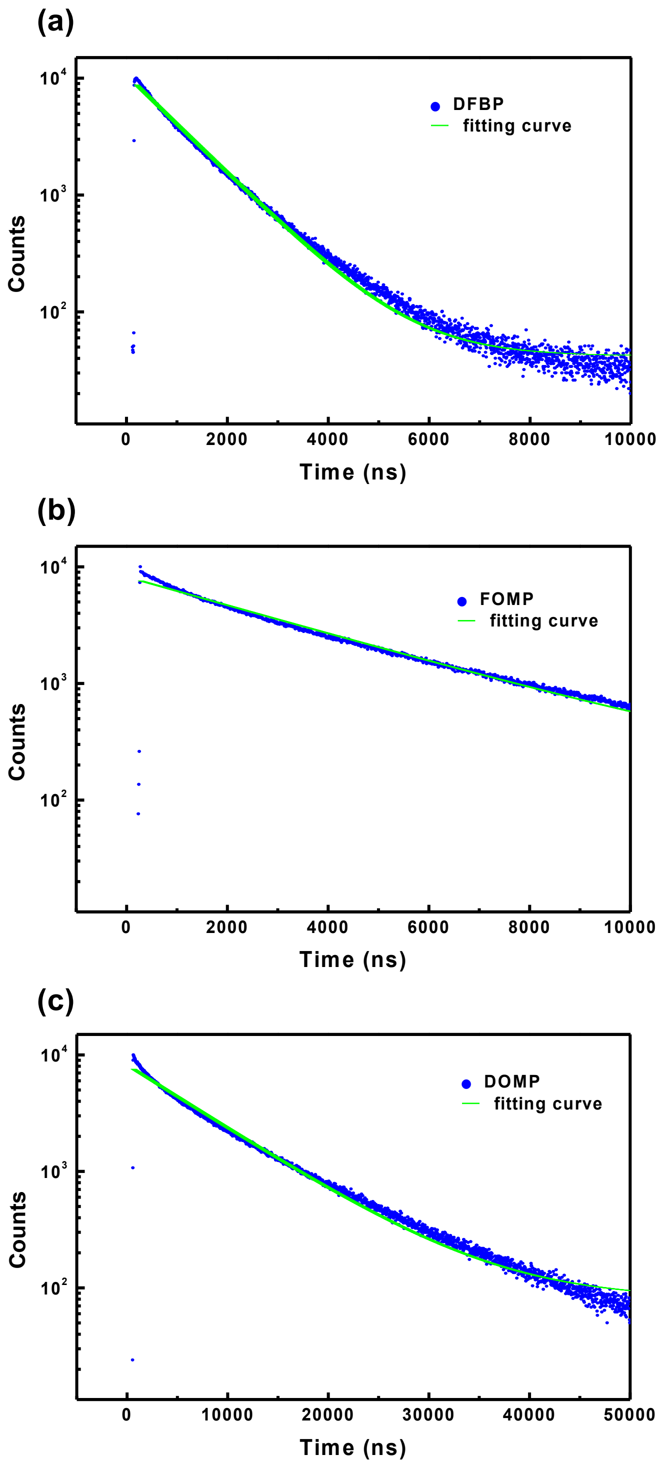

Figure S18. TrPL decay and fitting curve of (a) **DFBP**, (b) **FOMP**, and (c) **DOMP** in neat film at 298 K after excitation at 320 nm.

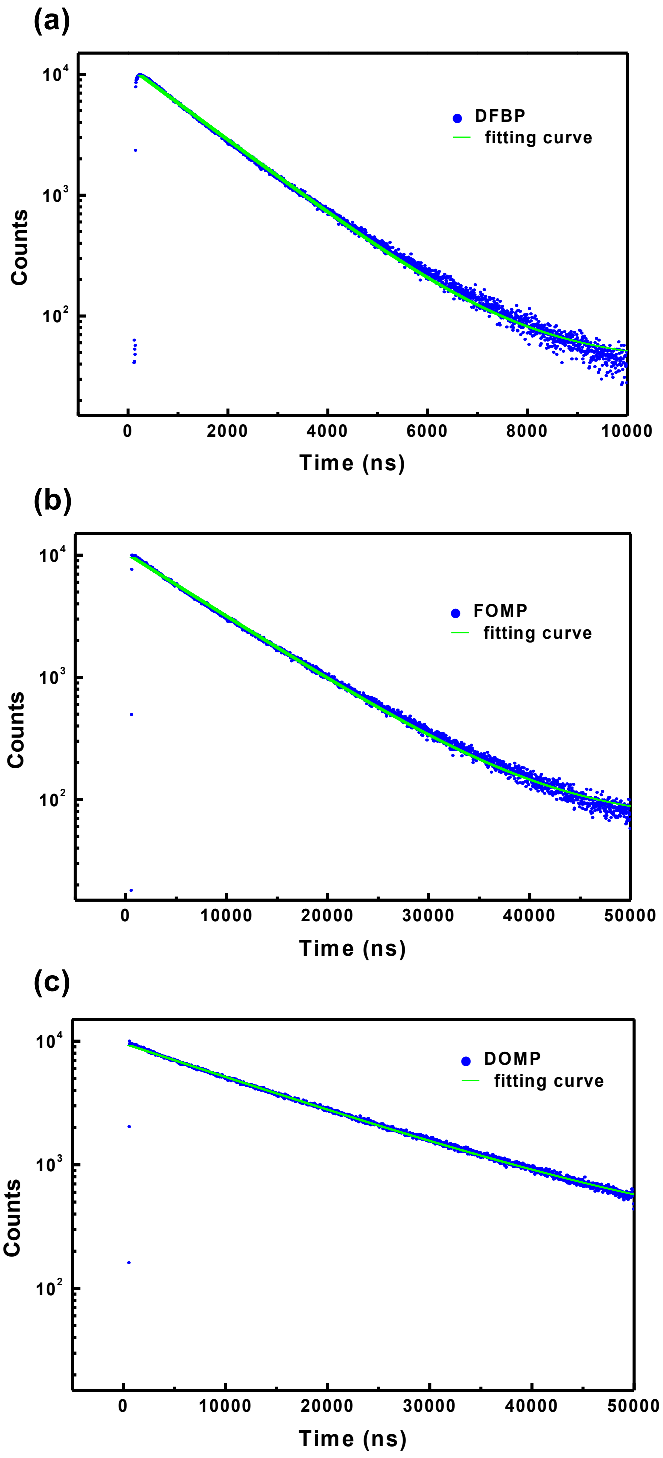

Figure S19. TrPL decay and fitting curve of (a) **DFBP**, (b) **FOMP**, and (c) **DOMP** in the thin-film contained complex (80 wt.%) and $[BMIM^+(PF_6^-)]$ (20 wt.%) at 298 K after excitation at 320 nm.

Thermal Analysis

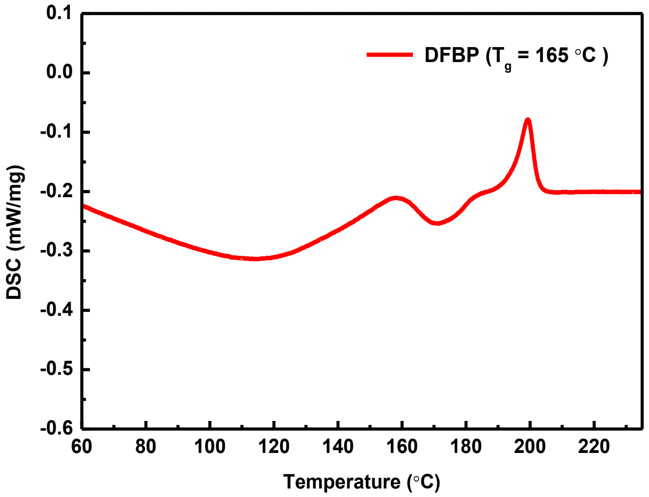

Figure S20. Differential scanning calorimetry (DSC) traces of **DFBP**.

Theoretical Calculation

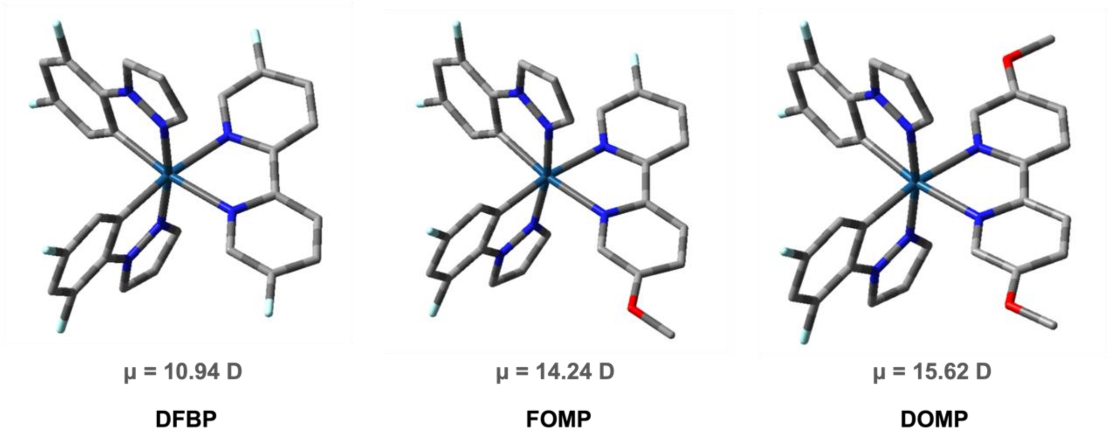

Figure S21. Optimized ground state geometry of **DFBP**, **FOMP**, and **DOMP** obtained in B3LYP/6-31G(d,p)/LANL2DZ level of theory.

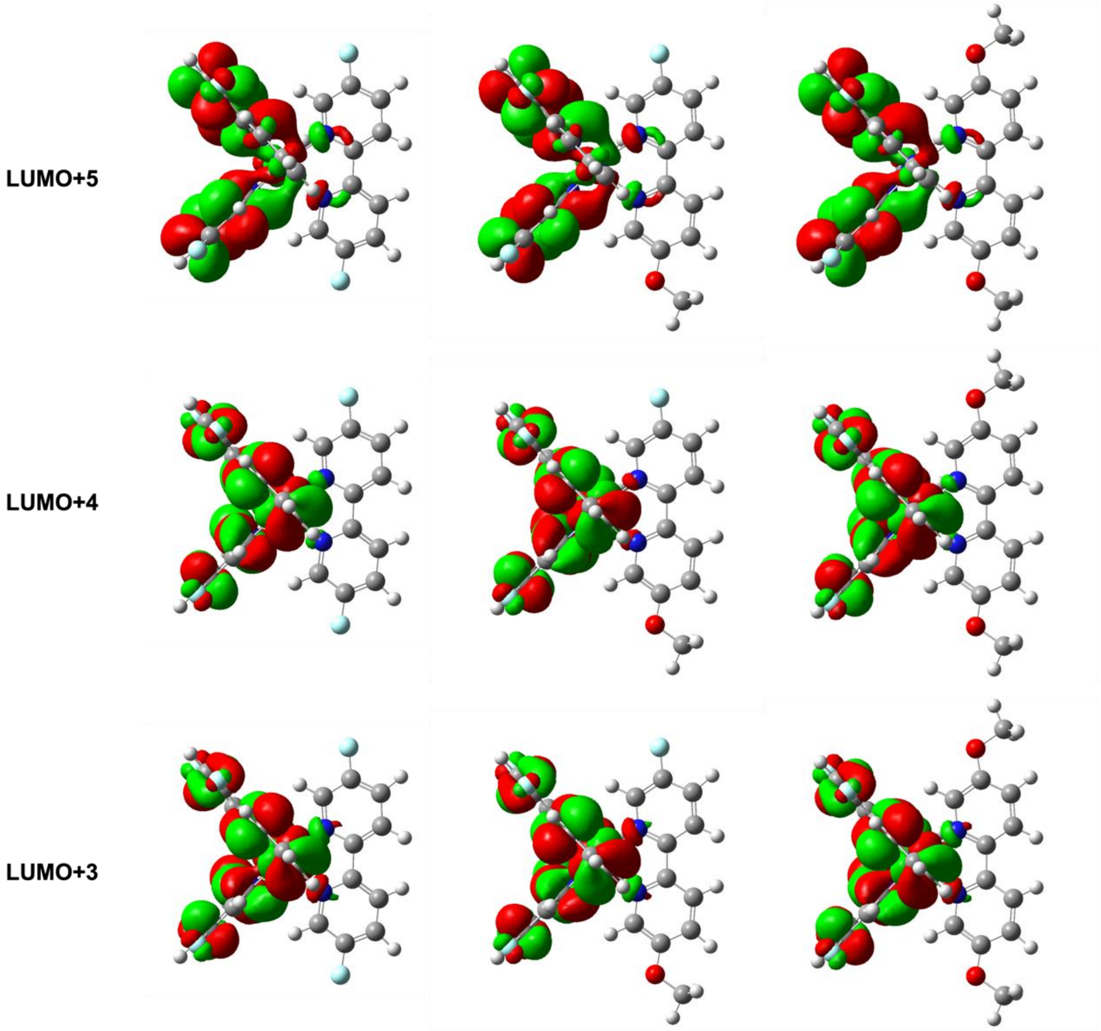

LUMO+2

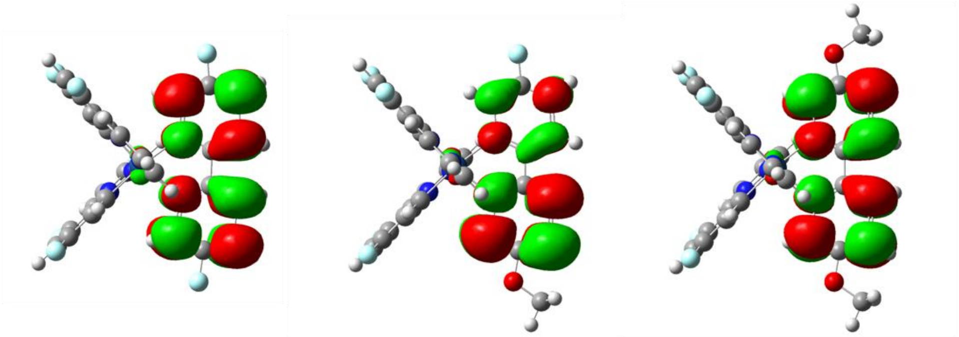

LUMO+1

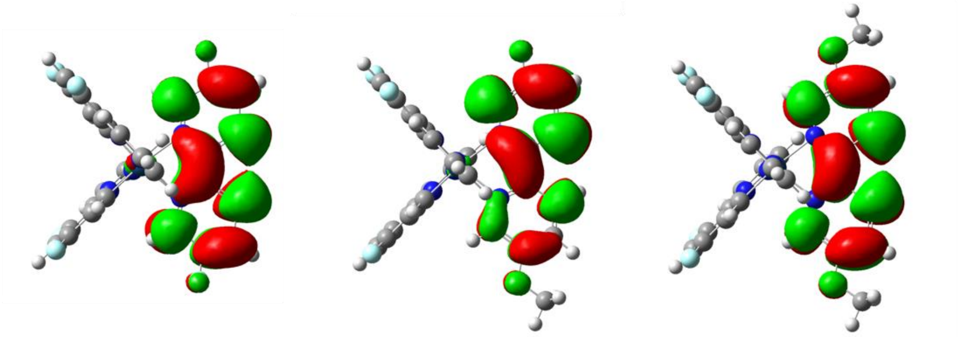

LUMO

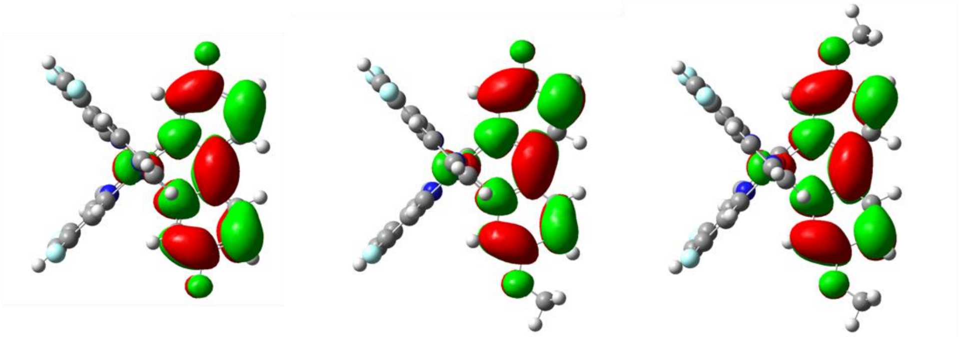

HOMO

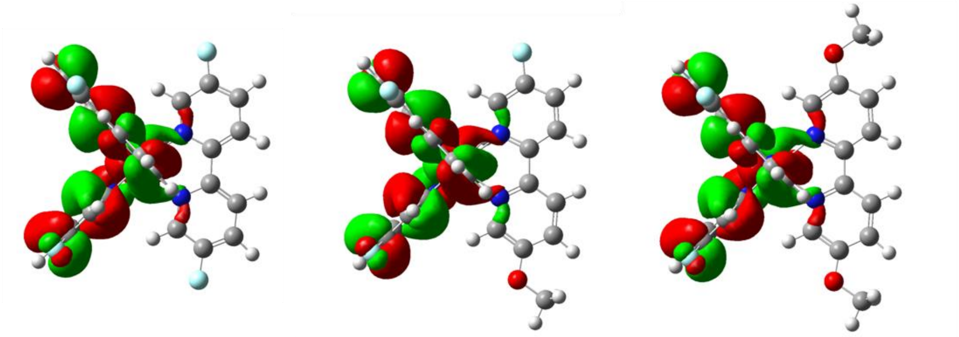

HOMO-1

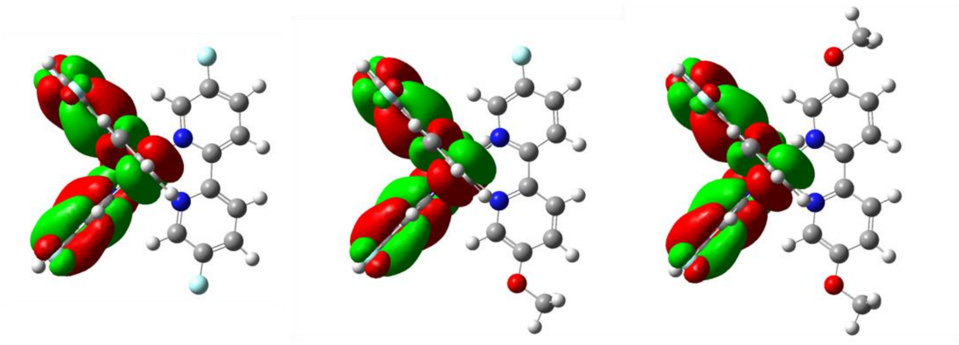

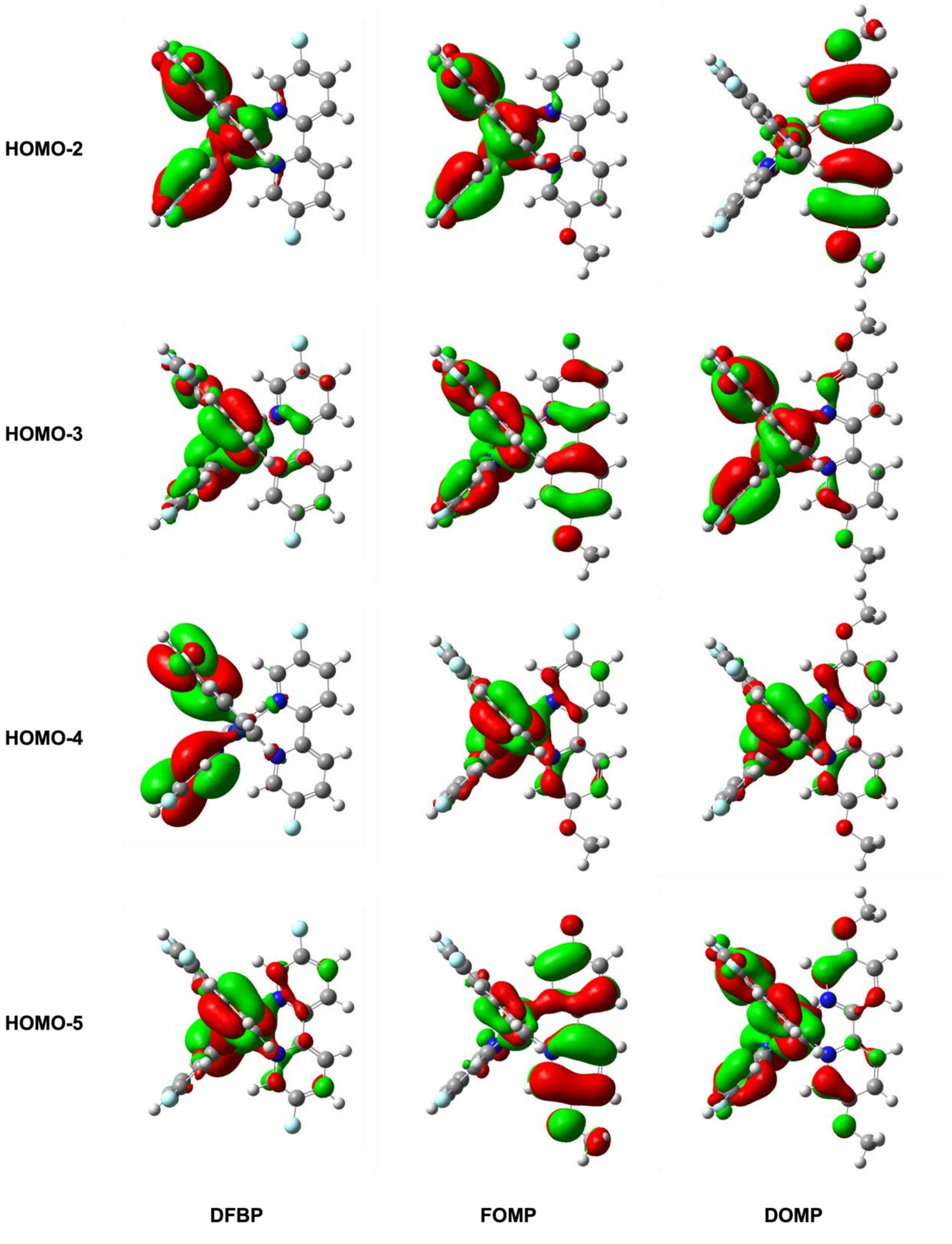

Figure S22. Frontier orbitals of **DFBP**, **FOMP**, and **DOMP** estimated by DFT.

Table S1. Selected TD-DFT calculations (B3LYP/LANL2DZ[Ir]6-31G(d,p)[F,O,N,C,H] basis set) of DFBP, FOMP, and DOMP.

Complex	States	eV	Dominant excitations
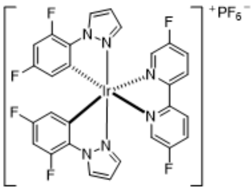 DFBP	S_1	2.53	H → L (97.2%)
	T_1	2.50	H → L (96.1%)<br>H-2 → L (2.3%)
	T_2	2.83	H-3 → L (61.1%)
	T_3	2.90	H-1 → L (49.0%)
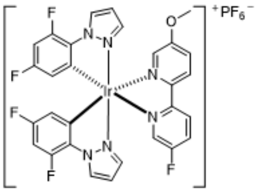 FOMP	S_1	2.78	H → L (97.3%)
	T_1	2.76	H-5 → L (27.4%)<br>H-5 → L+1 (5.2%)
	T_2	2.77	H → L (71.6%)<br>H-5→L (8.4%)
	T_3	3.14	H-4 → L (69.0%)<br>H-1 → L (19.4%)
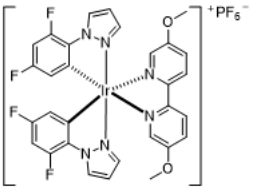 DOMP	S_1	3.05	H → L (97.5%)
	T_1	2.70	H-2 → L (80.0%)<br>H-2 → L+1 (6.7%)
	T_2	3.02	H → L (92.5%)<br>H-2 → L (2.6%)
	T_3	3.14	H-2 → L+1 (66.2%)<br>H-7 → L+2 (5%)<br>H-5 → L (2.6%)

Table S2. Summary of energies calculated for the frontier molecular orbitals (B3LYP/LANL2DZ[Ir]6-31G(d,p)[F,O,N,C,H] basis set).

Complex	HOMO [eV]	LUMO [eV]	E_g [eV]
**DFBP**	-7.76	-4.54	3.22
**FOMP**	-7.49	-4.01	3.48
**DOMP**	-7.25	-3.49	3.76

Table S3. Selected bond lengths in S_0 and T_1 states

Complex	State	$Ir\text{-}C_{(C\^N)}$ (Å)	$Ir\text{-}N_{(C\^N)}$ (Å)	$Ir\text{-}N_{(N\^N)}$ (Å)
**DFBP**	S_0	2.036	2.056	2.165
	T_1	2.038	2.055	2.161
**FOMP**	S_0	2.037	2.052	2.162/2.165
	T_1	2.039	2.047	2.131/2.142
**DOMP**	S_0	2.037	2.052	2.162
	T_1	2.041	2.050	2.133

EL Spectra

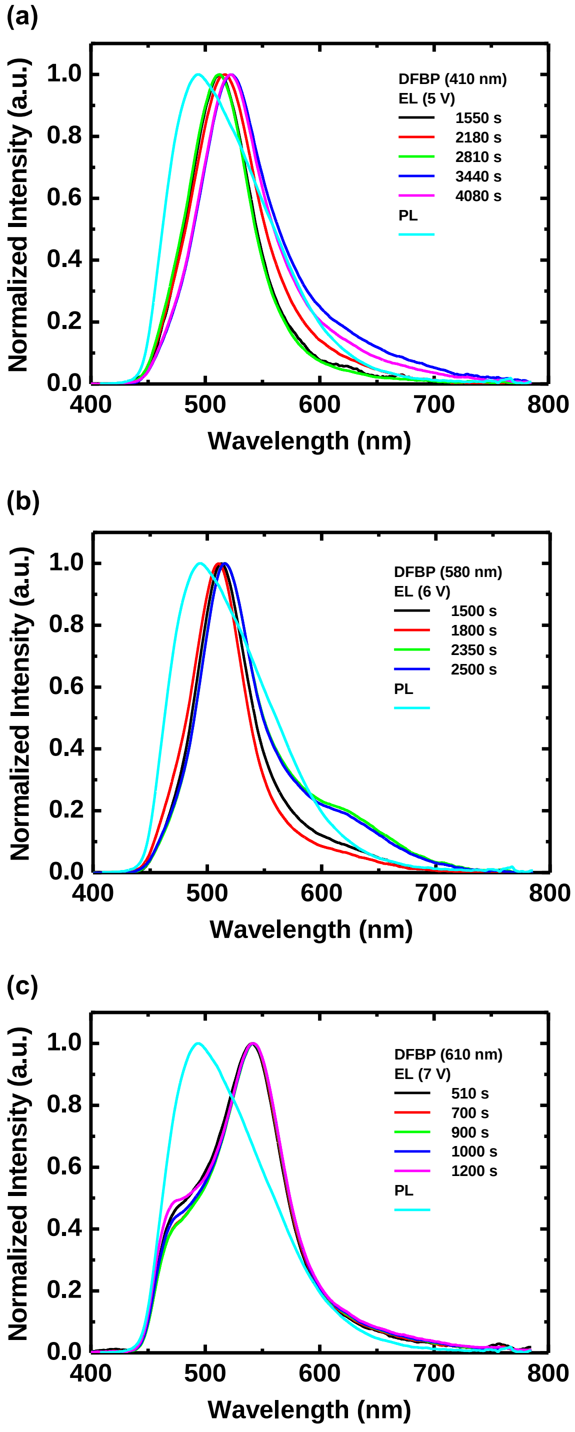

Figure S23. Time-dependent EL spectra of the LECs based on **DFBP** with emissive-layer thicknesses of (a) 410, (b) 580, and (c) 610 nm. The driving condition of each device is shown in the inset. The PL spectra of the emissive layers are also shown for comparison.

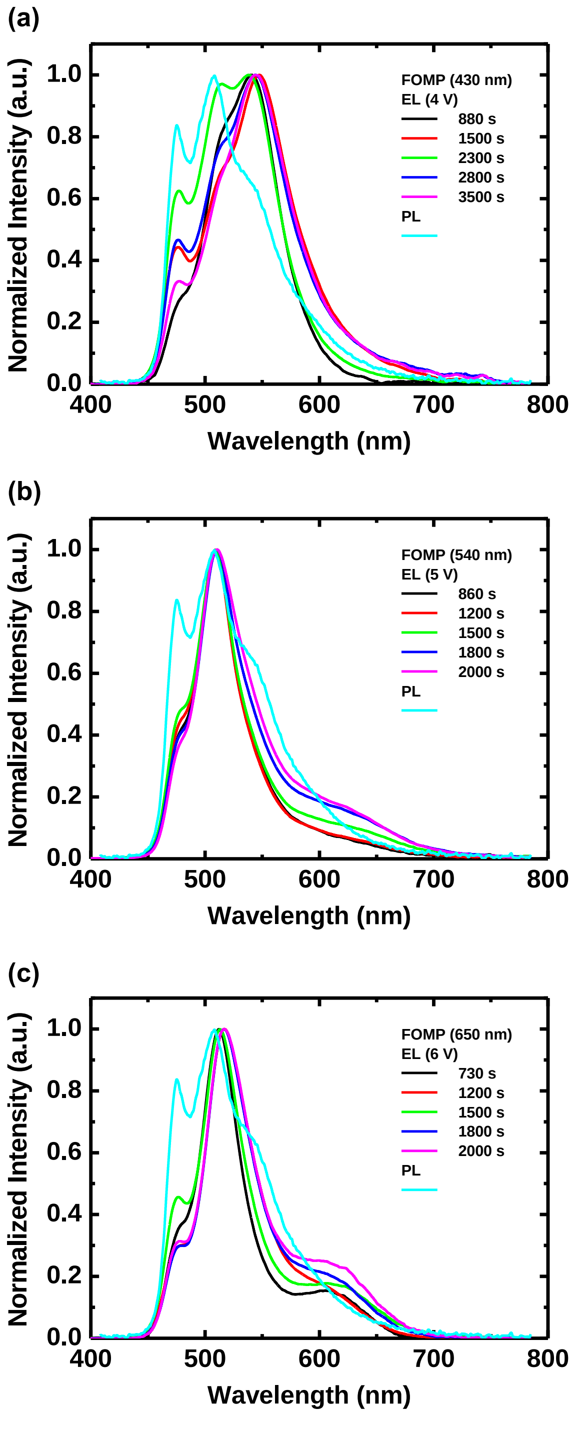

Figure S24. Time-dependent EL spectra of the LECs based on **FOMP** with emissive-layer thicknesses of (a) 430, (b) 540, and (c) 650 nm. The driving condition of each device is shown in the inset. The PL spectra of the emissive layers are also shown for comparison.

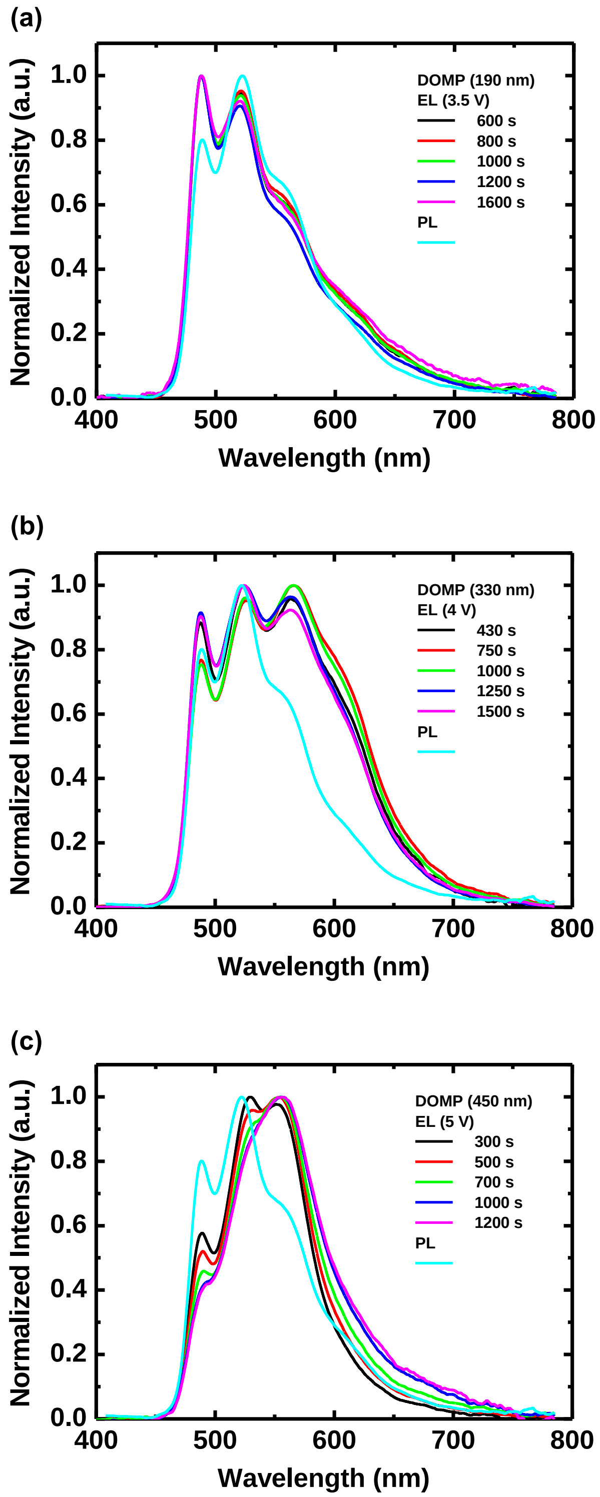

Figure S25. Time-dependent EL spectra of the LECs based on **DOMP** with emissive-layer thicknesses of (a) 190, (b) 330, and (c) 450 nm. The driving condition of each device is shown in the inset. The PL spectra of the emissive layers are also shown for comparison.

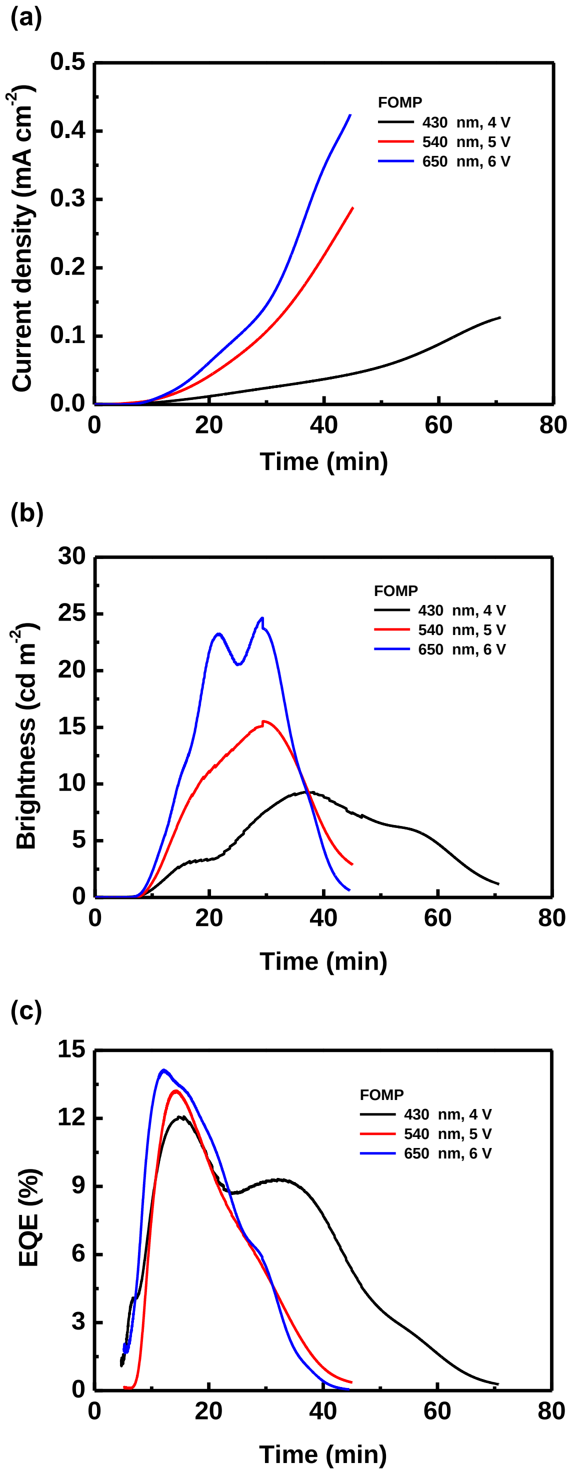

Figure S26. Time-dependent (a) current density, (b) brightness, and (c) EQE of the LECs based on **FOMP**. The driving conditions and device thicknesses are shown in the inset.

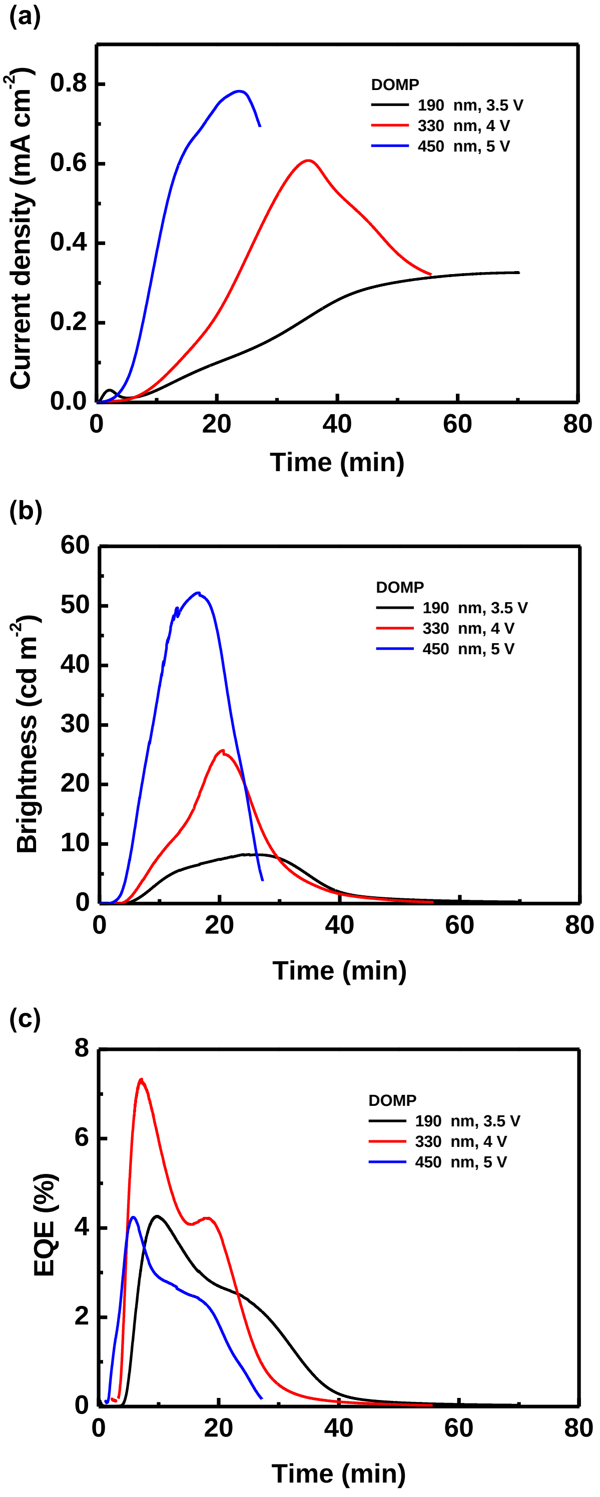

Figure S27. Time-dependent (a) current density, (b) brightness, and (c) EQE of the LECs based on **DOMP**. The driving conditions and device thicknesses are shown in the inset.

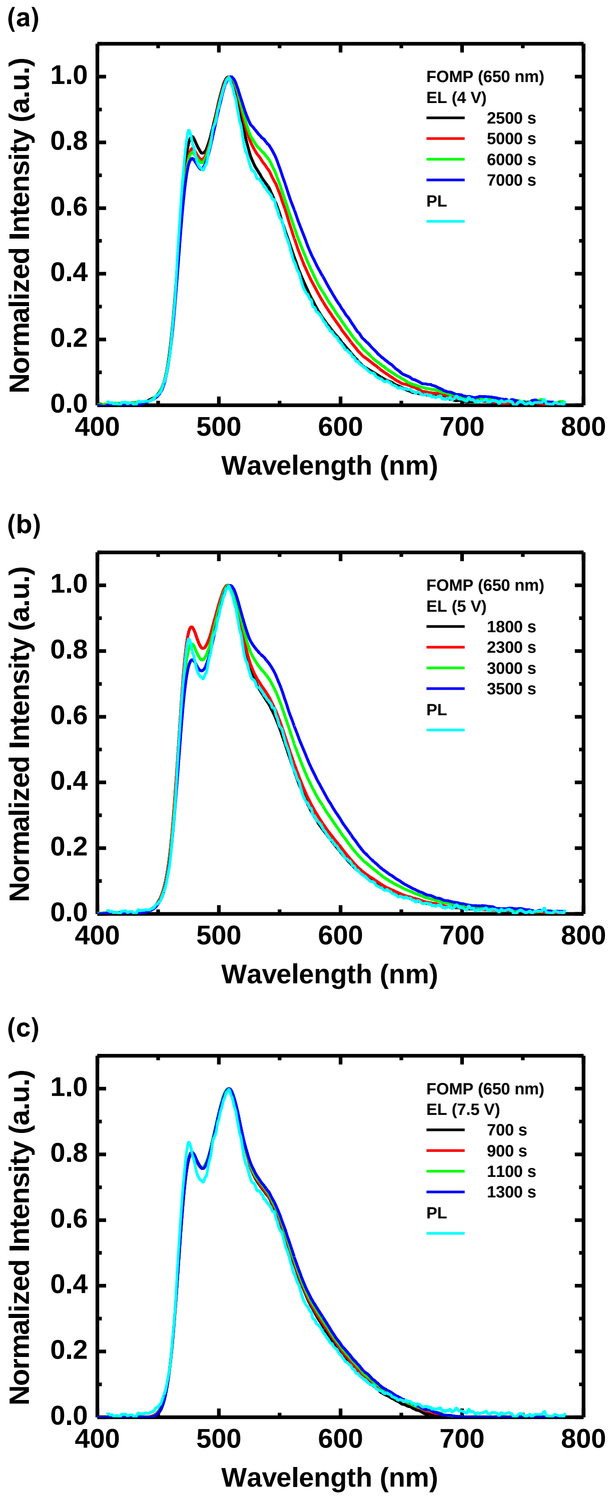

Figure S28. Time-dependent EL spectra of the optimized LECs based on complex **FOMP** (650 nm) integrated with the diffusive substrates under (a) 4, (b) 5, and (c) 7.5 V.

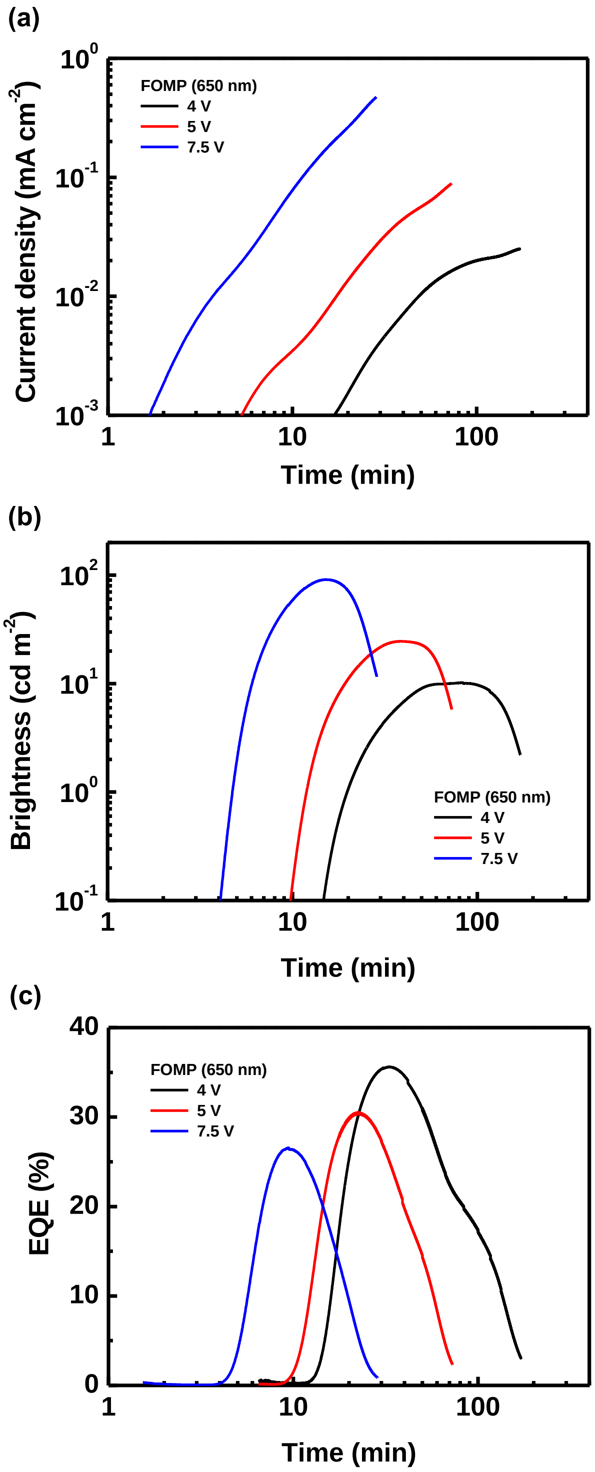

Figure S29. Time-dependent (a) current density, (b) brightness, and (c) EQE of the optimized LECs based on **FOMP** (650 nm) integrated with the diffusive substrates under 4, 5, and 7.5 V. The driving condition of each device is shown in the inset.

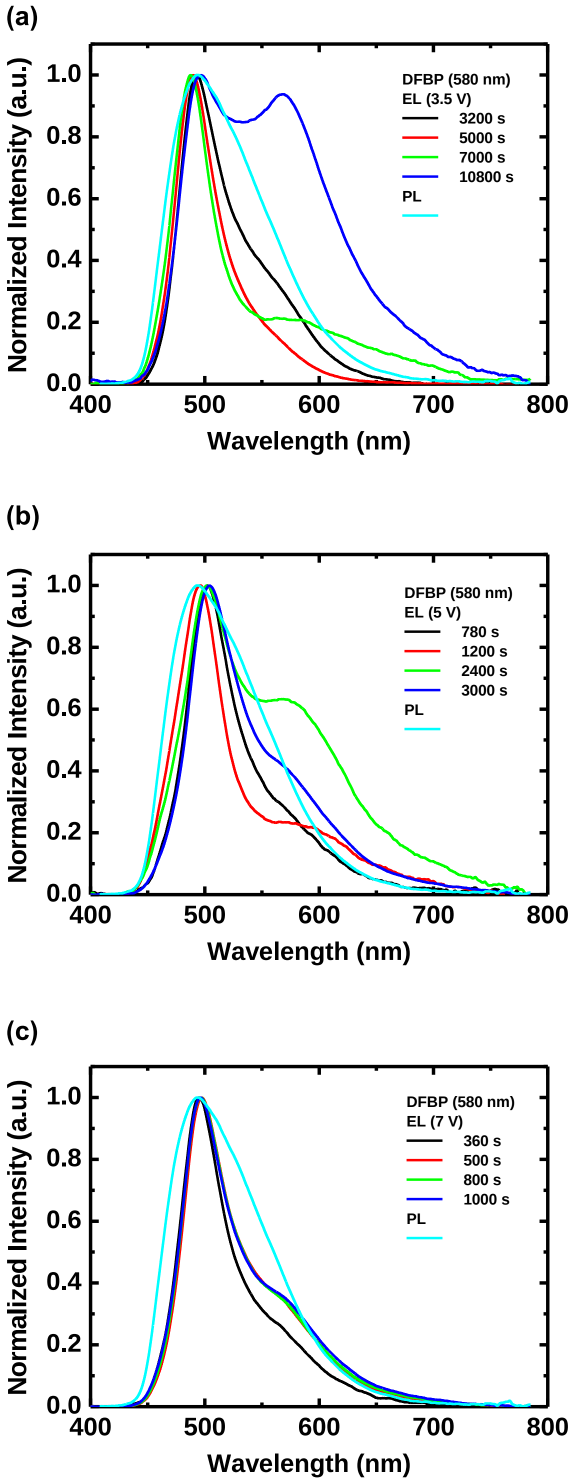

Figure S30. Time-dependent EL spectra of the reference LECs based on **DFBP** (580 nm) under (a) 3.5, (b) 5, and (c) 7 V.

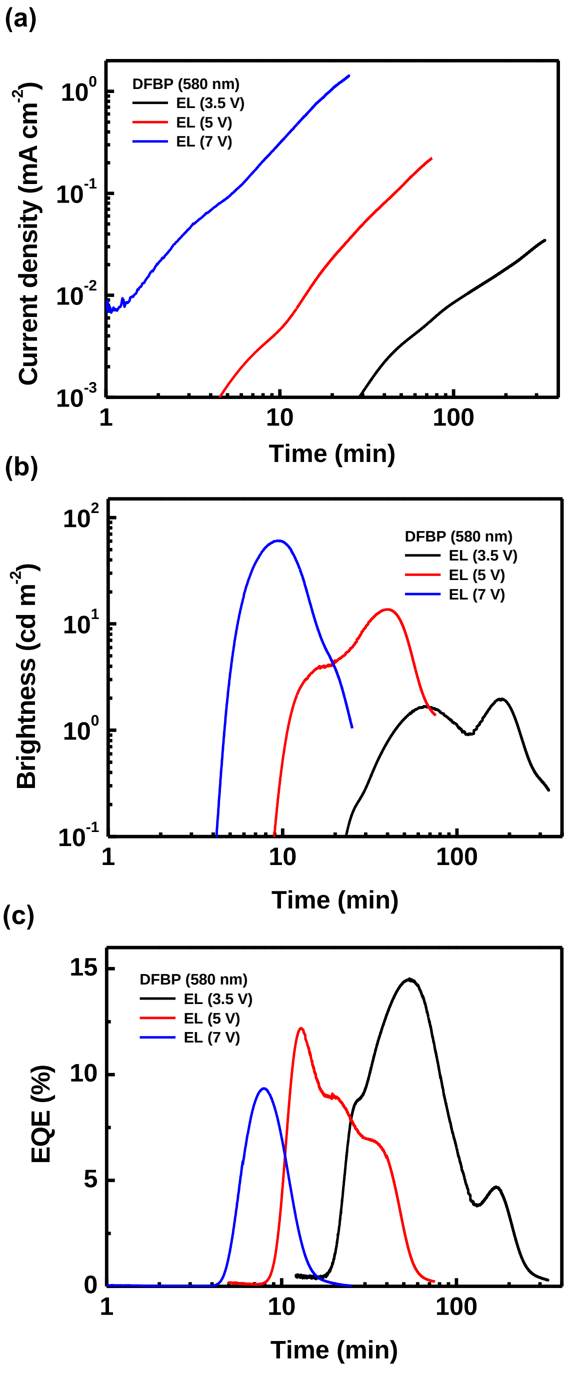

Figure S31. Time-dependent (a) current density, (b) brightness, and (c) EQE of the reference LECs based on **DFBP** (580 nm) under 3.5, 5, and 7 V. The driving condition of each device is shown in the inset.

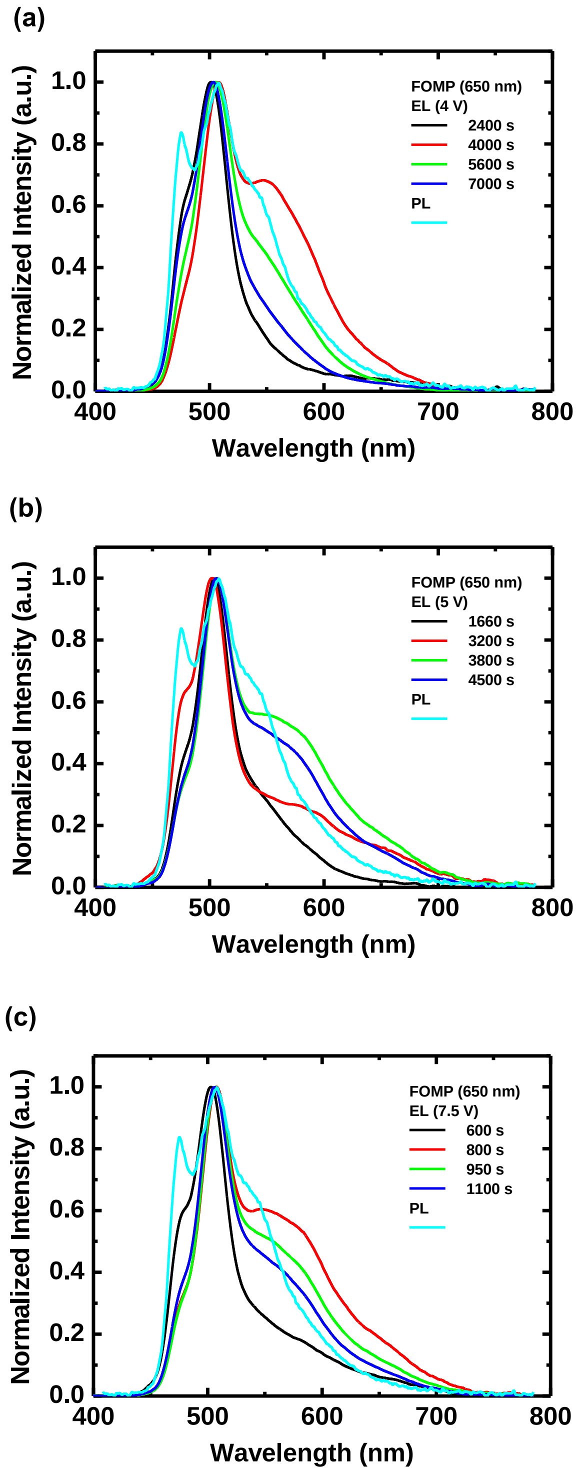

Figure S32. Time-dependent EL spectra of the reference LECs based on **FOMP** (650 nm) under (a) 4, (b) 5, and (c) 7.5 V.

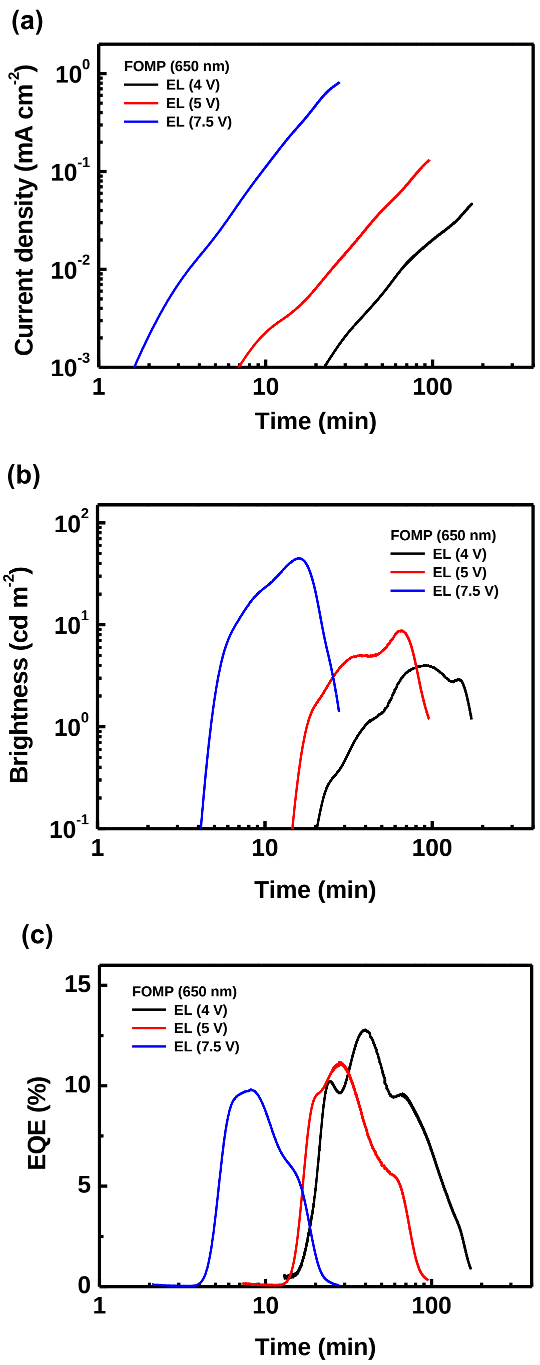

Figure S33. Time-dependent (a) current density, (b) brightness, and (c) EQE of the reference LECs based on **FOMP** (650 nm) under 4, 5, and 7.5 V. The driving condition of each device is shown in the inset.

Table S4. Comparison of the EL characteristics of the optimized LECs based on FOMP (650 nm) without and with diffusive substrates.

Device	Operation voltage (V)	EL_{max} (nm)[c]	B_{max} (cd m $^{-2}$)[d]	$\eta_{ext, max}$ (%)[e]	$\eta_{C, max}$ (cd A^{-1})[f]	$\eta_{P, max}$ (lm W^{-1})[g]
Without diffusive substrate[a]	4	504	4.0	12.8	29.7	23.4
	5	505	8.8	11.2	29.9	18.8
	7.5	506	44.8	9.8	23.6	9.9
With diffusive substrate[b]	4	510	10.2	35.6	98.3	77.2
	5	509	24.6	30.5	83.1	52.2
	7.5	508	91.2	26.6	76.9	32.2

[a] LECs fabricated on ITO (160 nm)/glass substrates; [b]LECs fabricated on ITO (160 nm)/diffusive layer/glass substrates; [c]EL emission peak wavelength; [d]Maximal brightness; [e]Maximal external quantum efficiency; [f]Current efficiency; [g]Power efficiency

Table S5. Summary of representative reported blue-green LECs emitters with EL peaks between of 470–535 nm and an EQE over 5%.

	Year	Complex	EL_{max} [nm]	$\eta_{ext, max}$ [%]	Ref.
1	2024	**DFBP**	516	16.8	This work
2	2024	**FOMP**	516	14.1	This work
3	2024	**DOMP**	524	7.3	This work
4	2024	**IMR-1**	503	13.0	[1]
5	2024	**IMR-2**	503	8.4	[1]
6	2024	**IMR-3**	494	8.3	[1]
7	2024	**DTBP**	522	10.3	[2]
8	2024	**DPPH**	534	9.2	[2]
9	2024	**FOMP**	523	7.1	[2]
8	2022	**H2**	474	8.6	[3]
9	2021	**Complex 1**	525	10.4	[4]
10	2021	**Complex 2**	517	7.2	[4]
11	2020	**Complex 4**	512	7.9	[5]
12	2020	**Complex 5**	500	5.5	[5]
13	2019	**Complex 2**	506	6.5	[6]
14	2019	**Complex 3**	488	6.3	[6]
15	2016	**Complex B**	499	9.9	[7]
16	2010	**$[Ir(dfppz)_2(tp\text{-}pyim)]PF_6$**	474	7.6	[8]
17	2008	**$[Ir(ppy\text{-}F_2)_2(dtb\text{-}bpy)]^+(PF_6^-)$**	525	14.9	[9]

Reference

(1) Zhang, K.; Pang, X.; Song, Y.; Xiu, Y.; Yu, R.; He, L. High-Performance Narrowband Light-Emitting Electrochemical Cells Enabled by Intrinsically Ionic Multi-Resonance Thermally Activated Delayed Fluorescence Emitters. *Advanced Optical Materials* **2024**, *12* (20), 2400467.

(2) Yi, R.-H.; Lee, Y.-H.; Huang, Y.-T.; Chen, X.-J.; Wang, Y.-X.; Luo, D.; Lu, C.-W.; Su, H.-C. Cationic Ir(III) Complexes with 4-Fluoro-4′-pyrazolyl-(1,1′-biphenyl)-2-carbonitrile as the Cyclometalating Ligand: Synthesis, Characterizations, and Application to Ultrahigh-Efficiency Light-Emitting Electrochemical Cells. *Inorganic Chemistry* **2024**, *63* (11), 4828-4838.

(3) Pang, X.; Zhang, K.; Song, Y.; Xiu, Y.; Yu, R.; He, L. Intrinsically-ionic donor-acceptor molecules featuring thermally-activated delayed fluorescence for high-performance host-guest blue light-emitting electrochemical cells. *Chemical Engineering Journal* **2022**, *450*, 137987.

(4) Yu, R.; Song, Y.; Chen, M.; He, L. Green to blue-green-emitting cationic iridium complexes with a CF3-substituted phenyl-triazole type cyclometalating ligand: synthesis, characterization and their use for efficient light-emitting electrochemical cells. *Dalton Transactions* **2021**, *50* (23), 8084-8095.

(5) Meng, X.; Chen, M.; Bai, R.; He, L. Cationic Iridium Complexes with 3,4,5-Triphenyl-4H-1,2,4-Triazole Type Cyclometalating Ligands: Synthesis, Characterizations, and Their Use in Light-Emitting Electrochemical Cells. *Inorganic Chemistry* **2020**, *59* (14), 9605-9617.

(6) Meng, X.; Bai, R.; Wang, X.; Pan, F.; He, L. Red to blue emitting cationic iridium complexes with 2-phenyl-4-dimethylaminopyridine as the cyclometalating ligand: Synthesis, characterization and electroluminescent devices. *Dyes and Pigments* **2019**, *165*, 458-466.

(7) Zeng, Q.; Li, F.; Guo, T.; Shan, G.; Su, Z. Large Size Color-tunable Electroluminescence from Cationic Iridium Complexes-based Light-emitting Electrochemical Cells. *Scientific Reports* **2016**, *6* (1), 27613.

(8) He, L.; Duan, L.; Qiao, J.; Dong, G.; Wang, L.; Qiu, Y. Highly Efficient Blue-Green and White Light-Emitting Electrochemical Cells Based on a Cationic Iridium Complex with a Bulky Side Group. *Chemistry of Materials* **2010**, *22* (11), 3535-3542.

(9) Bolink, H. J.; Coronado, E.; Costa, R. D.; Lardiés, N.; Ortí, E. Near-Quantitative Internal Quantum Efficiency in a Light-Emitting Electrochemical Cell. *Inorganic Chemistry* **2008**, *47* (20), 9149-9151.
